# A holistic view on the role of egg yolk in Old Masters’ oil paints

**DOI:** 10.1038/s41467-023-36859-5

**Published:** 2023-03-28

**Authors:** Ophélie Ranquet, Celia Duce, Emilia Bramanti, Patrick Dietemann, Ilaria Bonaduce, Norbert Willenbacher

**Affiliations:** 1 Institute for Mechanical Process Engineering and Mechanics, Karlsruhe Institute of Technology, Gotthard-Franz-Straße 3, 76131 Karlsruhe, Germany; 2 Consorzio Interuniversitario Nazionale per la Scienza e Tecnologia dei Materiali (INSTM), Via G. Giusti, 9, 50121 Firenze, Italy; 3 Department of Chemistry and Industrial Chemistry, University of Pisa, Via Moruzzi 13, 56124 Pisa, Italy; 4 Institute of Chemistry of Organo Metallic Compounds, CNR Via Moruzzi 1, 56124 Pisa, Italy; 5 Doerner Institut, Bayerische Staatsgemäldesammlungen, Barer Straße 29, 80799 Munich, Germany

**Keywords:** Lipid peroxides, Rheology, Colloids, Arts

## Abstract

Old Masters like Botticelli used paints containing mixtures of oils and proteins, but “how” and “why” this was done is still not understood. Here, egg yolk is used in combination with two pigments to evaluate how different repartition of proteinaceous binder can be used to control the flow behavior as well as drying kinetics and chemistry of oil paints. Stiff paints enabling pronounced impasto can be achieved, but paint stiffening due to undesired uptake of humidity from the environment can also be suppressed, depending on proteinaceous binder distribution and colloidal paint microstructure. Brushability at high pigment loading is improved via reduction of high shear viscosity and wrinkling can be suppressed adjusting a high yield stress. Egg acts as antioxidant, slowing down the onset of curing, and promoting the formation of cross-linked networks less prone to oxidative degradation compared to oil alone, which might improve the preservation of invaluable artworks.

## Abstract (web summary)

Old Masters used paints containing mixtures of oils and proteins, but we lack an understanding on how and why proteins were used. Here, the authors use egg yolk in combination with two pigments to evaluate how different repartition of proteinaceous binder can be used to control the flow behaviour as well as drying kinetics and chemistry of oil paints.

## Introduction

**Fig. 1:**
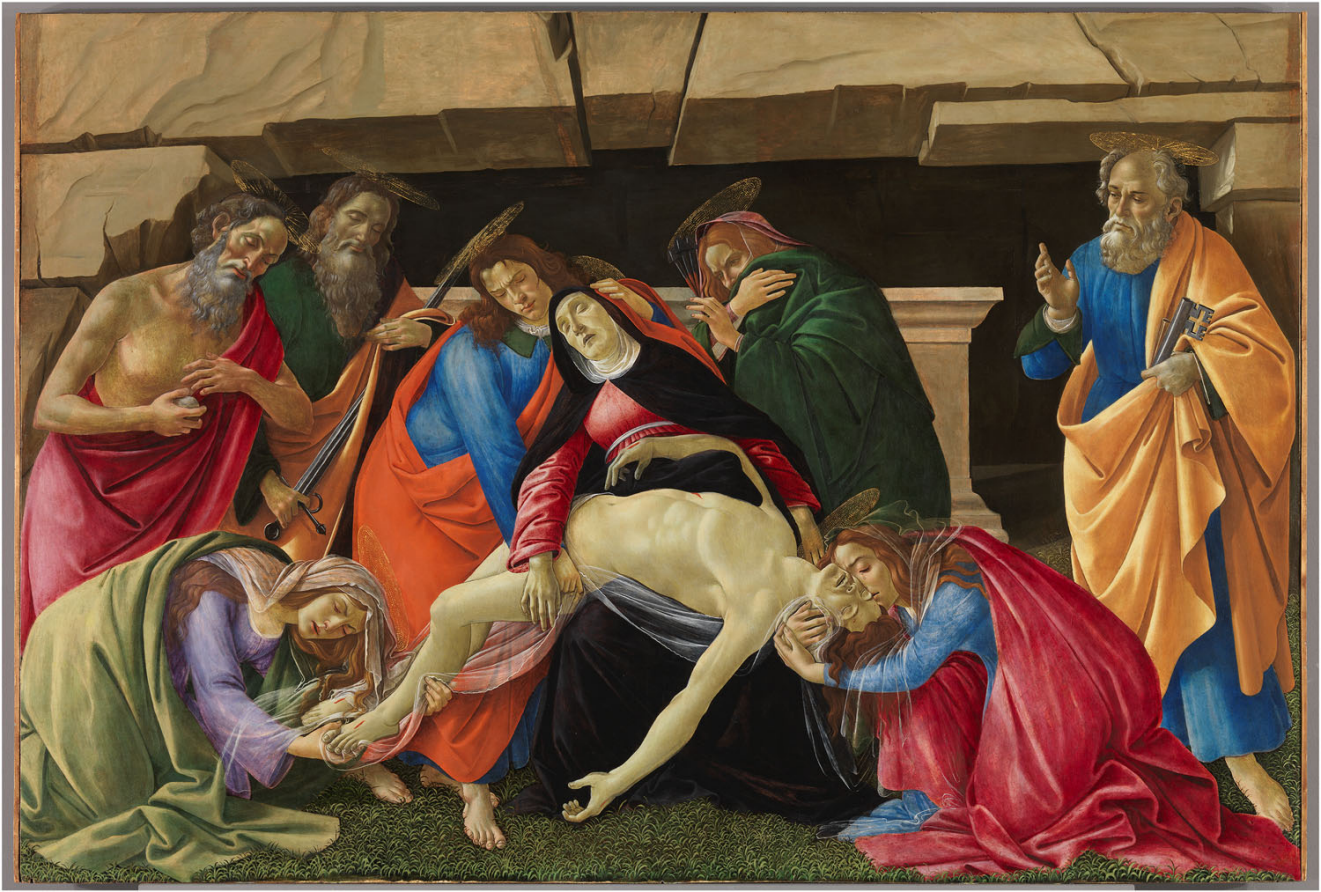
Sandro Botticelli, *The Lamentation of Christ*, c. 1490/95, 140 × 209.2 cm, Bavarian State Painting Collections, Inv. No. 1075. Although the flesh and some draperies are painted in egg tempera, the grass foreground and the stone tomb background are painted with oil paints containing proteins. © Bavarian State Painting Collections, Munich.

It is usually assumed that traditional Old Masters’ oil paints only contain oil as a binding medium, possibly with varying additions of some resins^1–4^. However, also proteins have been detected in oil paints by Sandro Botticelli (Fig. 1, Supplementary Tab. 1), Leonardo da Vinci and other Italian Renaissance masters^5,6^, as well as in Northern oil painting, e.g., late medieval Cologne paintings, and those by Albrecht Dürer, Johannes Vermeer, Rembrandt and many others^7–10^. So far it is difficult to decide where and when egg additives to oil paints have been used, because oil paints are often not analyzed for small amounts of proteins to minimize sample size, analytical time, and cost. However, the cited literature shows that such additives were identified in several centuries and countries, and they are not limited to specific pigments or layers.

What was the role of these proteins, how and why were they introduced into oil paints? The technical knowledge of the Old Masters, how paints had to be prepared, was initially passed down in workshops, but is lost today. It is known what pigments, binders, and techniques were used in general, but in insufficient detail. There is clearly more to the preparation of a paint than just mixing the pigment with a binder. For example, when in the 15th century Italian artists started to turn from egg tempera towards adopting Northern oil painting in one of the art-historically most important technical transitions in painting, they seemed to have had some problems, as the wrinkling in an early painting by Leonardo da Vinci testifies (cf. below). This is surprising, because oil paints had been known in Italy for more than 100 years: oil paints are described in Cennino Cennini’s famous treatise written c. 1400^11^, and oil binders have been identified in Italian 14th century paints^1,2,12^. However, these oil paints were usually monochrome colors applied on silver or gold leaf that did not need blending or complex paint application. Thus, oil painting seems to be more than painting with oil paints: protein additions might have been helpful to modify the oil paints’ properties in a beneficial way, allowing more sophisticated paint handling.

For painting, it is essential how liquid paints can be applied, whether they can be mixed wet-in-wet or not, and how they look after drying, which includes aspects like opacity and gloss, but also wrinkling or crack formation. Thus, flow properties of paints and the drying/curing of paint layers are crucial, which are correlated both to the chemical composition, and the colloidal microstructure of the wet paint. Sandro Botticelli used both egg tempera and oil painting techniques in some of his paintings^2,5^, as in the Lamentation of Christ (Fig. 1). Gas chromatography-mass spectrometry (GC/MS) and amino acid analysis (AAA) revealed mixtures of varying proportions of egg protein and oil in all six paint samples analyzed, most of them showing the appearance of oil paints (Supplementary Tab. 1)^5^, which confirms that other aspects than the exact composition are important for the classification of paints, as it was discussed for other types of paintings^13^.

But what possibilities of combining egg and oil did the Masters have, and what would have been the advantages with regard to rheology and stability of the resulting paints? Written sources such as Cennini do not describe the addition of proteinaceous materials to oil paints, but this is not surprising because the preparation of a pigment—a labor-intensive and time-consuming process—is often described in a different chapter, or not at all. The pigments, many of them deriving from minerals, had to be crushed and dispersed, cleaned and purified. There are many recipes describing the cleaning and preparation of pigments such as azurite or natural ultramarine, which sometimes involve addition of materials like glue, egg, but also polysaccharide gums or honey^11,14,15^. However, often these procedures are only mentioned for blue pigments and in context of painting techniques based on aqueous binders. The Liber diversarum arcium, presumably the most complete codification of medieval traditional oil painting, compiled c. 1300 and copied in Italy c. 1400, gives the only recipe according to our knowledge that is more explicit and directly links the addition of a proteinaceous binder to oil painting (recipe 1.3.9B)^14^: “[…] first grind with water and combine with three drops of glaire, and leave to dry in the sun, and once with time it is dried, repeat, and do this three or four times; afterwards temper it with oil [or] gum water, and use it.” Here we address the question whether such additions might also be useful for other pigments than the delicate and expensive blue pigments.

**Fig. 2:**
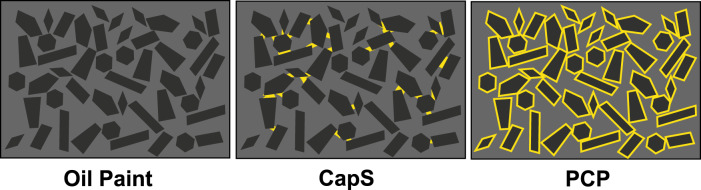
Proposed scheme of the paint model systems’ microstructure. The pigments are represented in black; the oil is in gray and the egg yolk is in yellow.

The cited recipe results in a protein coating of the pigments, before they are dispersed in oil, but various other systems may be formed with egg yolk or other aqueous proteinaceous solutions, drying oil and pigments, depending on paint preparation, as depicted in Fig. 2. Small amounts of egg yolk, mixed into an oil paint with a brush directly on the palette, can result in very stiff paints. This can be attributed to the formation of a fractal, percolating particle network induced by strong capillary forces acting in such a ternary system of solid particles and two immiscible fluids. In such systems, termed capillary suspensions, the immiscible secondary fluid phase forms pendular bridges (pendular state) or droplets incorporated into particle clusters (capillary state) which are much smaller than the suspended particles^16^. This sets capillary suspensions apart from emulsions where solid particles are added either to the continuous or disperse phase as well as from Pickering emulsions: in all these cases the particles are significantly smaller than the dispersed droplets^17^. Capillary suspensions, first described by Koos and Willenbacher^16^, have been observed for a wide variety of ternary solid/fluid/fluid systems^18–21^. If egg yolk, oil, and pigments are ground together with water, an oil-in-water emulsion is formed (not depicted in Fig. 2), which can be diluted with water. Such aqueous paints are labeled “tempera”, and, if oil is added the Italian term “tempera grassa” (i.e., fatty tempera) is used by technical art historians. Because tempera paints are significantly different from oil paints, particularly with respect to their drying behavior, they will be discussed in a subsequent paper.

Coating of pigments with proteins was said to stabilize the pigments preventing accelerated catalytic break-down of the oil binder and subsequent discoloration^7^. Beyond that, proteinaceous additives might have further advantages for many aspects of paint preparation, application, and drying.

In this work, we report the first systematic study on the effect of adding proteinaceous materials to oil paints. For simplicity, only egg yolk is considered here. We investigated oil paints made from protein-coated pigments and oil paints with a small amount of added egg yolk (capillary suspensions), to better understand Old Masters’ paintings, their techniques, and art historical developments. This research is seen as a proof of concept, demonstrating that such proteinaceous binders can be important additives. They can strongly influence the flow behavior of paints, i.e., their brushability and impasto, and hence the initial painting process. Egg yolk also affects the paints’ drying, i.e., the complex oxidation and chemical cross-linking process. Furthermore, it can affect the chemical and physical stability of aging paints, possibly reducing wrinkling and crack formation, yellowing, and darkening. This holistic view covers a wide range of different time scales of paint handling and aging, but also different length scales: from the molecular level, which determines oxidation and cross-linking, to the mesoscopic length scale of colloidal structures, which influence both wet paint flow and drying, to the macroscopic scale, which reflects brushwork and impasto, but also wrinkling and cracking. This provides new insights which may contribute to a better conservation and preservation of invaluable artworks.

## Results and discussion

Model paints were prepared using linseed oil (LO), a drying oil commonly used for artists’ painting^22^, egg yolk (EY) and either lead white (LW) or ultramarine blue (UB) as pigments (Supplementary Figs. 6 and 7). These pigments were selected for their extensive use in the artistic field through history and because they differently affect the curing and ageing of an oil paint layer^23^. Synthetic ultramarine blue was used instead of the natural one, available to Italian Renaissance artists. The choice was driven by the need of ensuring a constant pigment composition, which was necessary for the preparation of large batches of paints, replicated measurements, and several sets of systematic experiments that lasted about four years. It is well known that pigment shape and size distribution, exact chemical composition, and pre-treatment may all affect paint rheology, drying, and viscoelastic properties of dry paint layers^24,25^. As the focus of this work is on the effect of adding egg yolk to an oil paint, we selected two chemically very different, but readily available pigments to perform the comprehensive series of experiments and varied paint preparation only to achieve different repartition of egg yolk. The differently prepared model systems are schematically depicted in Fig. 2:

An oil paint consists of a pigment which is ground with linseed oil, to form a pure suspension.

A capillary suspension (CapS) is an oil paint in which a few drops of egg yolk are added and mixed together with a palette knife. The egg yolk triggers the formation of a percolating particle network^16,26^, resulting in a very stiff paint.

A protein-coated pigment (PCP), is prepared by first grinding the pigment with a solution of diluted egg yolk and subsequent drying. After evaporation of water, pigment particles coated with an egg yolk layer remain, and are then ground and dispersed with oil.

### Wet paint rheology

Rheological properties of paints have to be carefully adjusted to achieve the desired artistic expression. To paint impasto, i.e., with a visible, thick brushstroke, the paint must exhibit a sufficiently high yield stress preventing levelling after brush passage. Brushability, i.e., the force required to apply a paint layer, and the resulting layer thickness, are related to the high shear viscosity. These parameters can be easily changed by varying the pigment content^27^. Below we will discuss the complex effects of added egg yolk on these features depending on paint composition and preparation. Our first focus will be on impasto and yield stress, which is the critical stress required to break up the paint structure and enable flow. Yield stress determination and brushstroke analysis are described in the Supplementary Information.

**Fig. 3:**
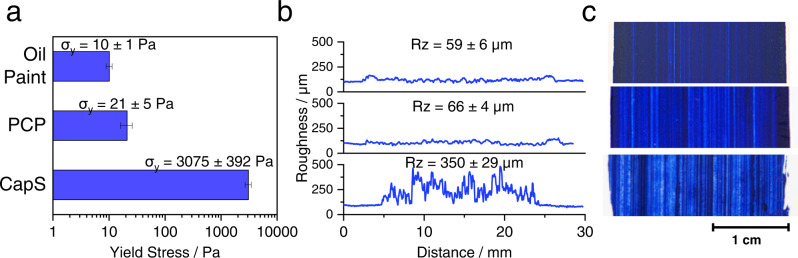
Wet paint yield stress and brushstroke profile. Ultramarine paints, relationship between **a** yield stress σ_y_, **b** roughness Rz and profile of the paint brushstrokes, and **c** appearance of the paint layer. The paints were prepared at a solid fraction of φ = 32 vol%, including 2 vol% of egg yolk solids for the paints containing proteins. The error interval denotes the standard deviation calculated from measurements performed at least in triplicate. PCP stands for protein-coated pigment paint and CapS for capillary suspension paint.

Paint formulations of identical solids volume fractions φ (including the pigment and in case of PCP also the egg yolk dry matter volume) were compared with respect to the brush profiles and roughness of dry paint layers obtained from automatically applied brushstrokes (Fig. 3).

The two pure suspension paint models (oil paint and PCP) showed similar, very regular paint profiles with low roughness Rz (<100 µm) in accordance with their low yield stress values (σ_y_ ≈ 10–20 Pa) at a solids content of φ = 32 vol%. The addition of 2 vol% of egg yolk as a coating of the UB pigment surface did not significantly modify the impasto. Yield stress data not only inform us about brushstrokes or impasto. They also provide valuable insight into the interactions among suspended particles and the strength and microstructure of the corresponding particle network^28–31^. The addition of the same volumetric amount of pure egg yolk into the oil paint as a secondary liquid phase leads to a strong increase of the yield stress (σ_y_ ≈ 3000 Pa). We attribute this to the formation of a percolating particle network induced by capillary forces inferred by the addition of a second, immiscible fluid phase, a generic phenomenon recently discovered and confirmed for a broad variety of colloidal systems^16,21,26^. Thus, levelling is prevented and the brushstroke profile exhibits strong fluctuation, i.e., the brushstroke is preserved with the CapS paint and this is a direct consequence of its orders of magnitude higher yield stress compared to the oil and PCP paints. Note, the capillary particle network is destroyed at high shear stresses σ >> σ_y_ and hence the high shear viscosity of the suspension is the same as for the corresponding binary suspension^26^, therefore the artist can apply the paint as easily despite the higher yield stress.

**Fig. 4:**
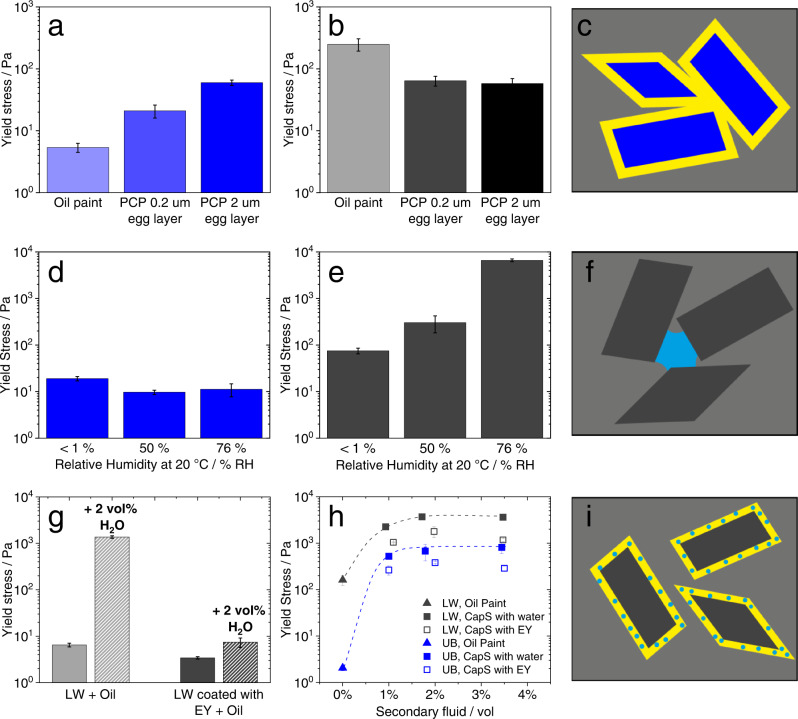
Influence of proteinaceous coating and humidity on yield stress and microstructure. Ultramarine blue (UB) paints are displayed in blue, and lead white (LW) paints in black. **a**, **b** Yield stresses of paints prepared at a solids fraction of φ = 32 vol% with or without an egg yolk (EY) coating, dispersed in linseed oil. The pigments were stored at a relative humidity of ≈50 RH% prior to be dispersed in oil. The thickness of the EY layer was calculated from the EY dry matter used to prepare the protein-coated pigments (PCP) assuming ideal monodisperse spherical particles and was included when calculating φ for the PCP paints. **c** representation of the steric attraction among pigment particles (in blue) due to the hydrophilic surface layers (in yellow), attempting to minimize the contact with oil molecules (in gray). **d**, **e** Yield stress of oil paints φ = 32 vol% prepared after a pigment storage of 2 weeks in humidity-controlled atmospheres prior to dispersion in oil. **f** Representation of the formation of capillary bridges between pigment particles (in gray) due to humidity (water in cyan). **g** Yield stress of LW oil paint φ = 18 vol% without (gray) and with EY coating (black), before and after addition of water as secondary fluid. **h** Yield stress of LW (black) and UB (blue) suspensions (triangle) and capillary suspensions prepared with EY (hollow square) or water (full square) as a secondary fluid, all prepared at a solid fraction of φ = 32 vol%. **i** Uniform distribution of water (in cyan) in the hydrophilic EY surface layer (yellow). The error interval denotes the standard deviation calculated from measurements performed at least in triplicate.

The presence of egg yolk (in PCP paints), has distinctly different effects on yield stress, depending on the pigment type. The yield stress increases in UB oil paints when the pigment particles are coated with egg, while it decreases in LW oil paints. Moreover, when increasing the amount of egg yolk in UB-based PCPs (Fig. 4a) the yield stress increases, but it does not significantly vary with EY content for LW-based PCPs (Fig. 4b). This different behavior can be rationalized assuming the egg layer essentially remains on the pigment (see Fig. 2 and Supplementary Fig. 6) also after dispersion in the oil since proteins are not oil soluble. Untreated, both pigments are very well wetted by the linseed oil (Ɵ_UB or LW-LO_ = 9 ± 2°), but the hydrophilic egg yolk coating leads to a significant increase in contact angle (Ɵ_EY-LO_ = 38 ± 7°), see Supplementary Tab. 2. For the UB-based paint, this results in an additional steric attraction^32^ among particles, due to the hydrophilic surface layers attempting to minimize the contact with oil molecules (Fig. 4c). Accordingly, the strength of the particle network, and hence the yield stress increases. Note, the hydrophilic character of the coating layer is decisive. A hydrophobic layer would act as a dispersing agent providing repulsive interaction leading to a reduction or complete elimination of the yield stress^17,32^. We are aware that a 2 µm protein layer on a pigment with 2 µm diameter is not a real artist paint, but such model systems are helpful to gain insight into the colloid-physical phenomena relevant for oil paints.

For the LW pigments another phenomenon prevails: the uptake of water from the ambient environment. Since external conditions (temperature, relative humidity) vary with the time of the day, the season and the geographical location, this challenges artists’ paint preparation and egg yolk may modify the effect of water adsorbed during pigment storage or paint application. We attribute the high yield stress of the LW oil paint to the formation of a CapS due to humidity uptake from the environment and we hypothesize, that the lower yield stress of the PCP is due to a uniform distribution of the absorbed water in the hydrophilic EY layer preventing the formation of CapS. Obviously, a 0.2 µm EY layer is sufficient to suppress this phenomenon. To further confirm this, the effect of humidity on paint yield stress was studied systematically.

Both pigments took up about the same amount of water when stored under controlled conditions of humidity and temperature (two weeks at relative humidity RH% of <1%, 50%, and 76%, and temperature T = 20 °C, for comparison the pigments used to determine the oil paint data shown in Fig. 4a, b were stored at a relative humidity fluctuating around 50%) prior to preparation of the oil paints (Supplementary Fig. 11). The effect on yield stress, however, is very different. For the UB pigment, water uptake does not affect the paint’s yield stress (Fig. 4d). In contrast, this quantity increases by almost two orders of magnitude for LW-based oil paints with an uptake of only 0.6 wt% water when stored at 76 RH% (Fig. 4e). Again, we attribute this to the formation of capillary suspensions similar as previously observed for other suspensions made from particles exposed to humid environment^21^. When exposed to the oil phase, the water on the particle surface creeps into the contact areas among adjacent particles and forms capillary bridges (Fig. 4f). This is obviously not the case for the oil paint made with UB pigment: in this case there seems to be no gain in free energy upon such a redistribution of water molecules. The phenomenon is further confirmed by adding 2 vol% of water as a secondary fluid to a φ = 18 vol% solids fraction LW oil paint, prepared with a previously dried pigment. Again, a capillary suspension with a yield stress two orders of magnitude higher than that of the corresponding oil paint is built (Fig. 4g). However, this phenomenon which may greatly disturb paint preparation in an artist’s workshop can be suppressed when the pigment surface is coated by a thin layer (≈0.2 µm) of proteinaceous material, e.g., egg yolk. In this case, the yield stress of the resulting PCP paint does not change significantly when adding the same amount of water. A capillary suspension does not form, presumably because the added water is uniformly distributed in the hydrophilic EY surface layer (Fig. 4g, i). Note, the pigments used to determine the oil paint data shown in Fig. 4a, b, were stored at a relative humidity fluctuating around 50%, and the corresponding yield stress data agree fairly well with those obtained using pigments stored at a controlled humidity of 40% for two weeks (Fig. 4d, e).

It is interesting to point out that capillary suspension paints can be formed if a sufficient amount of secondary fluid (either pure water, fresh EY, or another proteinaceous solution not miscible with the oil) is added to an oil paint, irrespective of the chemical structure and surface properties of the pigment. This phenomenon allows the yield stress to be varied over a wide range covering about three orders of magnitude (Fig. 4h). Samples containing fresh egg yolk exhibit slightly lower yield stress values than the ones containing pure water. This is attributed to the amphiphilic low-density lipoprotein complexes (LDLs) lowering the surface tension of the yolk compared to pure water, thus weakening the strength of the capillary particle network^33–35^. In fact, the interfacial tension between oil and water (Γ_LO-H2O_ ≈ 11 mN/m) is higher than that between oil and EY (Γ_LO-EY_ ≈ 2 mN/m), see Supplementary Tab. 3. The higher yield stress level of the LW CapS compared to the UB CapS is due to the larger fraction of fine particles in the LW samples^26^ (Supplementary Fig. 5).

In conclusion, the addition of egg yolk can have a strong impact on yielding of oil paints depending on the paint preparation procedure. A dry proteinaceous layer covering the pigment surface results in an attractive steric interaction among pigment particles which then leads to an increase in yield stress. Pigments can take up water during storage or paint preparation depending on humidity conditions, but water is also introduced when egg yolk is added to an oil paint. Then so-called capillary suspensions with their distinct percolating fractal particle network can form, resulting in very stiff paints. The yield stress can vary in a wide range depending on the amount of added secondary liquid and controlled by the wetting behavior of the aqueous phase in the ternary system, i.e., on its three-phase contact angle on the pigment surface and its interfacial tension with the oil phase. When the pigment particles, however, are coated with a hydrophilic proteinaceous layer, the water uniformly distributes in that layer and a capillary suspension does not form, leaving the yield stress unchanged.

In past centuries, artists may not have been able to control the humidity taken up by their pigments. In case the paint was too stiff, they presumably added more oil. However, if too much oil is added, binder-related problems arise, such as stronger discoloring (attributable to a more visible yellowing of the binder) and darkening (fatty acids released from ageing oil tend to react with lead white, forming metal soaps, which results more transparent paint layers), but also crack formation and even worse, wrinkling, a problem even Leonardo da Vinci encountered (see discussion below). Adding some proteinaceous material during pigment preparation, resulting in a coating layer, might have solved the problem of unintentional formation of capillary suspensions, resulting in better, more stable paints with higher pigment content. This surface treatment not only prevents the formation of capillary suspensions but also leads to a reduced increase of high shear viscosity with increasing solids content and hence allows for better brushability at high pigment loading (Supplementary Figs. 8 and 9).

### From wet paints to solid films

Several complex mechanisms may control film formation. Egg proteins are commonly employed to form films and coatings in various products such as food or pharmaceuticals^35,36^. Evaporation of solvent in protein-based paints results in dry films.

The drying of an oil paint layer is a chemically driven process, which entails the oxidation and cross-linking of the constituting polyunsaturated triglycerides (curing). As the curing reactions progress, the oil gradually becomes viscous, tacky, and finally dry to the touch, which is a process that may take from hours to several days (Supplementary Fig. 10), allowing the artists to work on the impasto, mix the paints on the palette and work with wet-in-wet technique. Drying might be influenced by several factors^23,37,38^, such as the environmental conditions (light, temperature, humidity) and the intrinsic properties of the film such as its chemical composition (binder and pigment) and the layer thickness^39,40^.

We investigated how the natural air-drying of linseed oil paints is affected by the egg addition. In the curing of a drying oil, upon exposure to air, oxygen is added, with consequent formation of peroxide species, initiating the curing reactions^37,41,42^. Peroxide species may be detected and estimated with differential scanning calorimetry (DSC) and thermal gravimetry (TG).

**Fig. 5:**
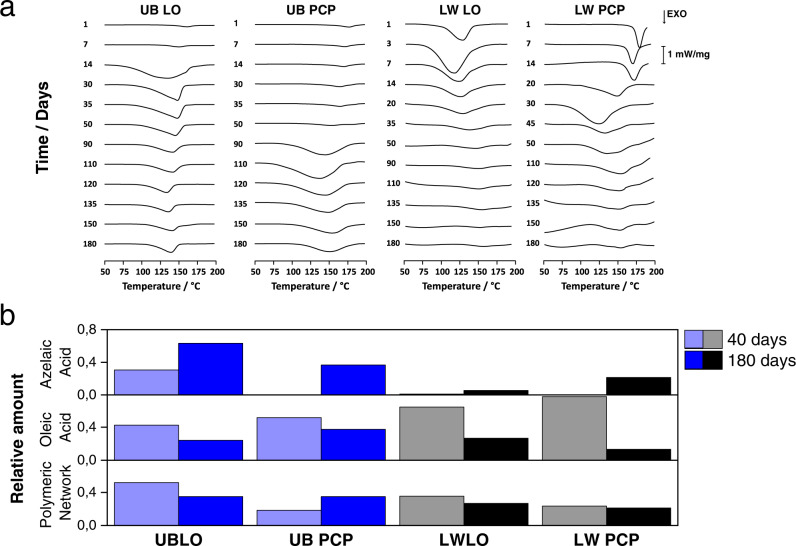
Curing of oil paint layers. DSC curves (**a**) and relative amount Py/GC/MS extracted ion pyrograms (**b**) of ultramarine blue (UB) and lead white (LW) model paints up to 180 days of natural ageing. Solids content of UB LO, UB PCP, LW LO, and LW PCP are φ = 6, 33, 31, and 29 vol%, respectively, including 12 vol% (UB PCP) and 15 vol% (LW PCP) of EY. **b** The evolution of the oil polymeric network (*m*/*z* 129), the azelaic acid (*m*/*z* 317), and the oleic acid (*m*/*z* 339) of the different formulations at the same curing time. All DSC curves are normalized to the oil content and all the relative amount of the pyrolysis products to the palmitic acid peak *m*/*z* 313. PCP stands for protein-coated pigment paint and LO for oil paint.

In a DSC experiment a sample collected from a paint layer is progressively heated under inert atmosphere, and in the temperature range 75–200 °C, an overall exothermic peak is observed, which is due to two main thermal effects. The first, endothermic, consists of the decomposition of hydroperoxides and peroxides formed by the action of oxygen on the triglycerides. The second, exothermic, is related to the recombination of radical species, both deriving from the decomposition of the same peroxides and hydroperoxides, and those formed upon the curing process. The area of the overall peak is proportional to the amount of peroxides and active radical species present in the paint at the moment of the experiment^23^. DSC curves are shown in Fig. 5a, Supplementary Figs. 13 and 14.

In a TG experiment we observe a mass loss in the temperature range corresponding to the DSC peak, due to the thermal decomposition of peroxide species.

The formation of peroxides is also visible by monitoring the mass change of a paint layer over time. In particular, upon curing a mass increase is observed due to the addition of oxygen, with formation of peroxides. With time, other reactions follow, some of which may lead to the formation of small molecular weight species, which may evaporate from the paint film, leading to a mass decrease. We compared the gravimetric mass increase (wt%) with the mass loss observed in TG experiments in the temperature range corresponding to the DSC peak ^43^. We could detect the presence of active radical species in linseed oil samples right before the onset of mass uptake of the macroscopic samples, see Supplementary Fig. 12.

**Fig. 6:**
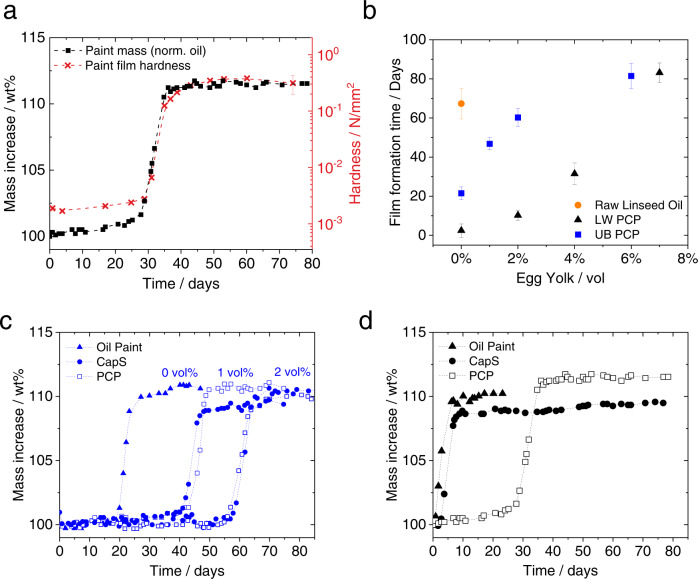
Curing kinetics and film formation of oil paints. **a** Displays the comparison between the mass uptake (black square) of a protein-coated paint film (LW PCP at 4 vol% of EY), normalized to its oil content, over time and the corresponding change in mechanical hardness (red cross). The error interval denotes the standard deviation calculated from measurements performed at least in triplicate. **b** Film formation time (the time at which the paint has reached its maximum weight change) vs. egg yolk content for PCP-type paints; error bars represent the interval between the start and the maximum of the weight increase. **c**, **d** Mass increase, normalized to the oil content, of UB (blue) or LW (black) paint samples prepared as oil paints (triangle), capillary suspension CapS (circle) or PCP (square) paints as a function of time. **c** UB CapS and PCP paints contain 1 and 2 vol% of EY solids. **d** LW CapS and PCP paints contain 4 vol% of EY solids. All studied paints contained a solids fraction (pigment and EY solids) of φ = 32 vol%.

Simultaneously, we measured the hardness of the paint surface at regular time intervals pushing a cylindrical piston into the layer at constant speed and depth, imitating the dipping of a finger into the paint. When the paint is still liquid, the hardness of the paint remains low (<0.01 N/mm^2^), but this value increases up to two decades upon film formation and approaches a plateau, which characterizes the “dry-to-touch” state of the paint film when a solid film is formed. The mechanically determined “dry-to-touch” transition clearly corresponds to the steep increase of the sample mass indicating the onset of oxidation and chemical drying (Fig. 6a). This has been suspected earlier, but quantitative mechanical data were lacking^41^.

The addition of pigments into raw linseed oil speeds up its drying kinetics: a 0.4 mm thick layer of raw linseed oil takes up to 70 days to dry, and this time is reduced to 20 and 4 days for paints including 32 vol% of UB and LW pigments (but no egg yolk), respectively (Fig. 6b). Indeed, LW acts as a catalyst for curing reactions and thus allows for shorter drying times compared to the UB paints^23,44^.

When adding increasing amounts of EY as pigment coating, we observe a delay in the curing reactions and drying time (Fig. 6b). This is also evident from the DSC (Fig. 5a and Supplementary Fig. 13), TG (Supplementary Fig. 12) and gravimetric data both at natural (Figs. 5a and 6c, d) and accelerated ageing at 80 °C (Supplementary Fig. 19). This can be ascribed to the antioxidant properties of egg yolk, which are exerted by phospholipids, representing 10 wt% of the yolk dry content, phosvitin, proteins, and free aromatic amino acids, tending to inhibit oxidation of lipids by different mechanisms^45^. Moreover, egg yolk contains carotenoids and vitamin E, which are lipophilic compounds preventing lipid per-oxidation by either radical transfer or by stabilization of peroxyl radicals^45^. Such stabilization of peroxyl radicals is also well evident from the onset temperature of the DSC peroxide decomposition/radical recombination peak. Supplementary Figs. 15 and 16 and Supplementary Tab. 4 show that the presence of egg shifts the onset temperature during the first days of natural aging especially for the PCP paints with their high EY content. With time, the onset temperature of the peak tends to reach the same value for all systems (Supplementary Fig. 15), possibly because upon hardening of the paint layer, the reaction pathways might be more strongly affected by the mobility and chemical reactivity of active species present in the oil phase, away from the oil/pigment and oil/protein interfaces.

For UB-based paints, the latency time of drying is the same for CapS and PCP containing the same amount of EY solids (Fig. 6c): the antioxidant effect of egg is exerted irrespective of the protein repartition in the microstructure of the UB paints. For the LW paints, however, the EY distribution has a significant impact: when EY is restricted to the small contact regions between adjacent particles in the capillary suspension paints, the drying time is close to that of the oil paint. In LW PCP paints, however, the drying time is longer (Fig. 6d). If the assumption that the protein coating does not change with drying and curing is correct, this difference observed in the UB and LW paints may be explained by the fact that the catalytic effect of LW is exerted upon the dissolution of lead into the oil^46^. In CapS, most of the pigment particle surface is in direct contact to the oil phase, making it possible for the lead to dissolve into the oil and catalyze the curing reactions. As a result, LWLO and LW CapS behave very similarly from the curing kinetics point of view. In PCP the EY is evenly distributed on the pigment surface, serving as an “antioxidant shield”, delaying the dissolution of lead into the oil, and possibly affecting the products of reaction between the metal and the binders. This feature has been used earlier to prevent the lipid peroxidation of linseed oil in o/w emulsions^47^. PCP-type LW paints, however, are faster to start the curing process than UB ones, but the differences tend to disappear when large amounts of egg yolk are added (Fig. 6b). Our data show that CapS takes up less oxygen than PCP at given egg yolk content. As the interfaces oil/protein/pigment are different in the two systems we may expect a change in the oxidative reaction pathways and kinetics.

The delay in the onset of the oil curing observed when EY is added, and the higher stability of peroxides produced, entails a delay in the formation of oxidation products of the oil as well as the oil polymeric network. This shows up in the analysis of oil paint films at different stages of curing by analytical pyrolysis coupled with GC/MS (Py/GC/MS) (Fig. 5b and Supplementary Figs. 17 and 18). At 40 days, oil paint layers already show an extensively cross-linked oil network, a relatively high content of azelaic acid (stable product of oxidation), but the unsaturated oleic acid is still present, indicating the curing is not complete. With ageing, the amount of azelaic acid increases, and that of oleic acid decreases, furthermore the relative content of the polymeric network slightly decreases with time, due to oxidative degradation phenomena, as reported previously^23^. In PCP-type UB paints at 40 days the relative content of the polymeric network is very small, and increases steadily with time. On the other hand, and in agreement with the catalytic activity of LW, in LW PCP the cross-linked network is well present at 40 days and appears very stable over time. Concurrently the relative content of azelaic acid increases with time and that of oleic acid decreases. The data available at this stage seem to indicate that when egg is present oxidative degradation occurs to a smaller extent. TG experiments under accelerated conditions at 80 °C confirm that egg preserves the oil paint from oxidative degradation: the mass loss is small also after several hours of accelerated curing in UB PCP and LW PCP compared to UBLO and LWLO (Supplementary Fig. 19).

The addition of egg thus seems to favor the formation of a well cross-linked network. This might be due to the known reactivity between lipid oxidation products and proteins^48–50^, which may cause polymeric network modifications in the paint^51^ and co-polymerization^52^. Indeed, egg proteins respond to the chemical environment generated by the oxidizing lipids in a paint layer as shown with secondary structure analysis of the protein component in PCP systems by peak fitting of the amide I band of TRANS-FTIR spectra^53^. CapS were not studied with this technique as the amount of added EY is below the detection limit. Previous research on model systems based on combinations of different pigments and proteinaceous materials showed that proteins undergo structural modifications which depend on the nature of the pigment and the age of the paint layer^54^. Our results here show that the relative content of beta structures, helix and random coils of proteins in PCP systems is subject to significant changes in correspondence to the maximum of the mass increase upon curing (Supplementary Fig. 20), when the concentration of peroxide species is very high (Supplementary Fig. 12).

The experiments on the drying/curing of the paint layers stressed out that formulations containing EY presented longer drying times compared to oil paints. These particularly long drying periods are unrealistic for artists’ paintings, and inconvenient, but may be overcome by applying other well-known remedies, such as drying agents, or heat treatment of the oil^11,44^. On the other hand, the addition of proteinaceous materials seems to produce well cross-linked polymeric fractions, less prone to oxidative degradation, which may lead to conservation issues^37^.

### Wrinkling

**Fig. 7:**
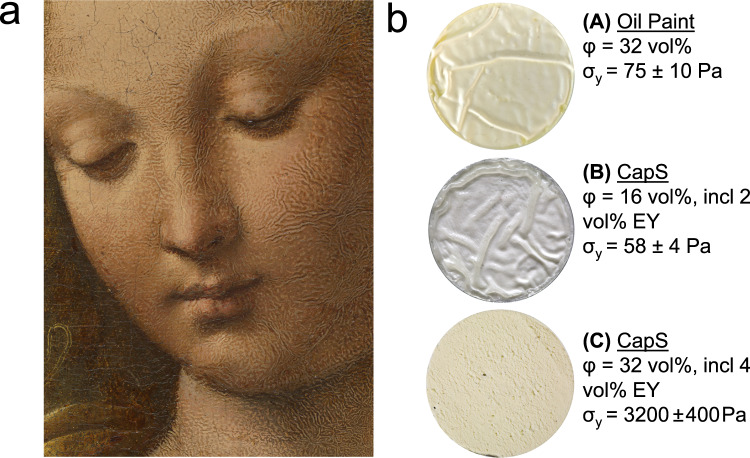
Wrinkling and yield stress of paints. **a** Detail of Mary’s face displaying wrinkling of the flesh paint in Leonardo da Vinci’s *Madonna of the Carnation*, c. 1475, Inv. No. 7779, 62 × 48.5 cm, © Bavarian State Painting Collections, Munich, the whole painting is shown in Supplementary Fig. 22. **b** Photographs of LW paint surface structures after film formation (thickness = 1.0 mm) depending on pigment content φ and yield stress σ_y_. CapS denotes capillary suspension and EY denotes egg yolk.

Wrinkling may occur during the transition from the wet paint to the dry layer, and may deteriorate the quality and appearance of a painting. Wrinkling occurs rather quickly, within days, and thus areas were usually removed and improved by artists. As a result, examples of paintings showing wrinkling are rare. One of them is Leonardo da Vinci’s *Madonna of the Carnation*, c. 1475, which shows extensive wrinkling on the flesh of Mary (Fig. 7a and Supplementary Fig. 22).

When linseed oil cures, a film may form at its surface due to a gradient of oxygen distribution in the paint layer, resulting in an uneven drying between the surface and its bulk. Because oxidation results in a volume increase of the oil, the surface area can be enlarged by wrinkling, as long as the oil below the dried skin is still liquid. This effect depends on the difference of film formation rate between the surface and the deeper layers^55^, which is especially strong if oils with very high drying rates are used, such as tung oil or linseed oil with too much added catalytic drier^56,57^, or they are applied in too thick layers^55^ (see also Supplementary Fig. 23). It is also well known that wrinkling is more likely to occur in paints containing too few pigments^44^. Increasing the solids fraction in oil paints thus prevents the formation of wrinkles (Supplementary Fig. 24). The phenomenon is, however, not simply related to pigment loading.

Wrinkling of oil paints may also be avoided adding egg to the paint formulation. In Fig. 7b, the oil paint with 32 vol% solids content exhibits similar wrinkling as the CapS with only 16 vol% particle loading adjusted to the same yield stress level (≈ 58–75 Pa) adding 2 vol% EY. However, wrinkling can be suppressed when the mobility of the paint below the dry skin is drastically reduced as shown here for the oil paint with 32 vol% solids with 4 vol% EY added, thus increasing the yield stress to σ_y_ ≈ 3200 Pa. The wrinkling phenomenon is tightly related to the paints’ yield stress and can be strongly reduced by thoughtfully adjusting this material property. Adding egg yolk to oil paints is a skillful method to achieve this, especially when a high pigment loading is to be avoided, e.g., for reasons of artistic expression. This new insight may encourage further targeted investigations regarding the composition of paint layers with and without wrinkles.

This holistic study combines knowledge from conservation science, rheology, and analytical chemistry to understand in which various ways Old Masters like Botticelli, da Vinci or Rembrandt might have used proteinaceous binders to modify oil paints to create their artworks. Exemplarily using two major pigments employed for painting during many centuries, lead white and ultramarine blue, we discuss various ways to produce different microstructures (protein-coated pigments vs. capillary suspensions) and how this can be used to control the flow behavior of paints, as well as their drying kinetics and mechanisms according to the artist’s requirements. It is shown how artists might have used proteinaceous materials to influence impasto of their fresh oil paints, to overcome unexpected problems with humidity, produce paint layers stable against wrinkling and oxidative degradation, giving us the opportunity to admire their masterpieces still today.

## Methods

Ingredients: Model paint formulations included up to four ingredients (pigment, oil, fresh egg yolk, and distilled water). We used either lead white (Kremer Pigmente, Germany) or synthetic ultramarine blue (Blu Oltramare Puro M (6018), Abralux Colori Beghè, Italy) as pigments with an average size x_50_ = 2 µm, but slightly different size distribution (Supplementary Fig. 5). The referenced densities are 2.35 g/cm^3^ for UB and 6.7 g/cm^3^ for LW. Cold pressed linseed oil (Maimeri, Italy) was used without further treatment, for ageing experiments it was sampled from freshly opened bottles and stored at room temperature conditions in a closet. The environmental conditions were fixed (temperature at 20 °C, relative humidity 30–50%, and light exposure). Fresh organic hen’s eggs were purchased in local supermarkets and used no longer than 3 days after purchase. Typically, a fresh hen’s egg yolk contains 17 wt% of proteins, 31 wt% of lipids, 2 wt% of carbohydrates and ashes, and around 50 wt% of water^58^. The water content of each egg was measured with a moisture analyzer (HX204, Mettler Toledo) to adapt the egg yolk solid content to each formulation).

The solids fraction was 32 vol% for all paints with some documented exceptions and the thickness of the paint layers was 0.4 mm. A description of the experimental methods can be found in the Supplementary Information, and a movie for the paint preparation is included (Supplementary Movie 1).

## Supplementary information

Supplementary Information

Description of Additional Supplementary Files

Supplementary Movie 1

## Data Availability

Due to the complexity of the multidisciplinary topic, the data that support the findings of the study is available upon request.
