# Supplementary material for: A holistic view on the role of egg yolk in Old Masters’ oil paints
Source: Nat Commun. 2023 Mar 28;14:1534. doi: 10.1038/s41467-023-36859-5 (PMC10050151; doi:10.1038/s41467-023-36859-5)
Supplement: Supplementary file 1 — Supplementary Information [file 41467_2023_36859_MOESM1_ESM.pdf]

Supplementary Information

A holistic view on the role of egg yolk in Old Masters' oil paints

Ophélie Ranquet*, Celia Duce, Emilia Bramanti, Patrick Dietemann*, Ilaria Bonaduce*, and Norbert Willenbacher*

1. Egg yolk and oil in a historic artwork

Sandro Botticelli's Lamentation of Christ

Supplementary Table 1. Results of binding media analyses of Sandro Botticelli's Lamentation of Christ, Bavarian State Painting Collections, Inv. No. 1075, published in Dietemann et al. 2017.[1] For sample positions A and B cf. Supplementary Figure 1. The classification of paints into oil and tempera has been performed by an interdisciplinary team of conservators and scientists mainly based on the appearance of the paints, which include optical and rheological aspects (cf. Supplementary Figures 2-3) as well as knowledge from sources and chemical analysis. +++ large quantity, ++ intermediate quantity, + small quantity, (+) very small amount.

Area	Paint layer	Classification	Oil (GC/MS)	Protein (AAA)	Contaminations from varnish
A: Green meadow on red dress of mourner on the right	Light green grass	Oil	++	(+)	Linseed oil, dammar, mastic, copaiba balsam
	Yellow-green grass	Oil	++	++	
	Dark green background of grass	Oil	+++	+	
	Red from dress, with white highlight layers	Egg tempera	+++	+++	
B: Stone of tomb	Dark grey paint of shadow	Oil	++	++	Dammar, mastic, Strasbourg turpentine, copaiba balsam, beeswax, linseed oil
	Light brownish paint of stone	Probably oil	+++	++	

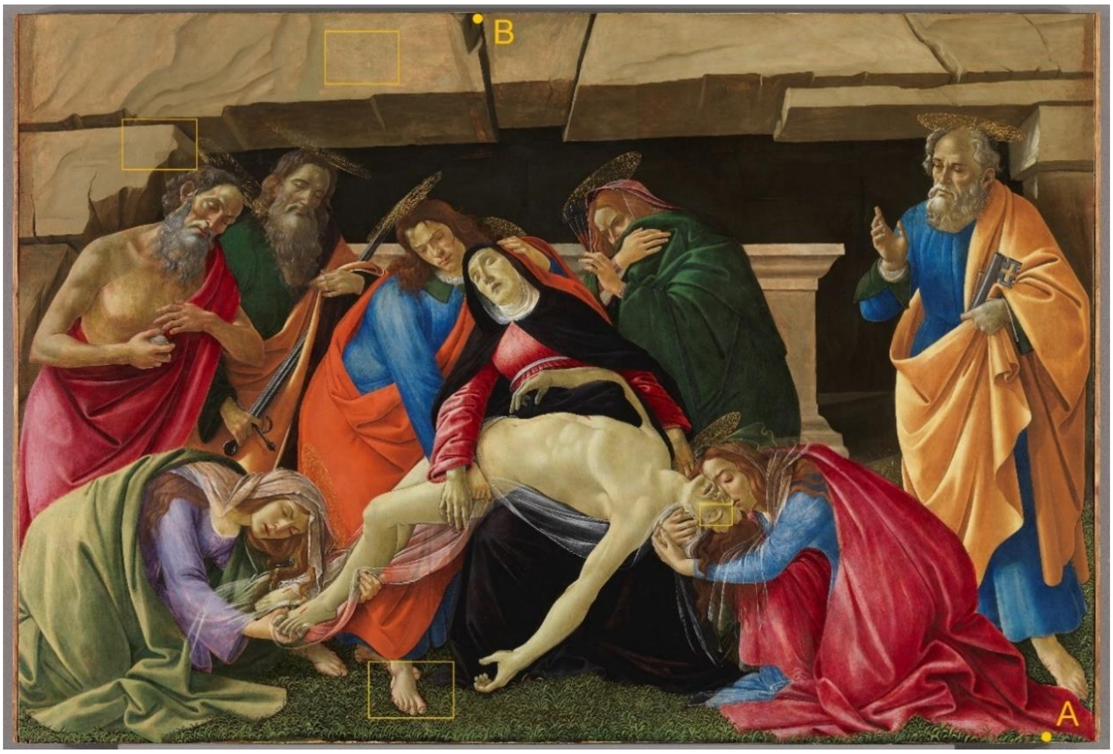

Supplementary Figure 1. Sandro Botticelli, *the Lamentation of Christ*, with indication of sampling positions for binding medium analyses reported in Supplementary Tab. 1, and details displayed in Supplementary Fig. 2-3. © Bavarian State Painting Collections, Munich.

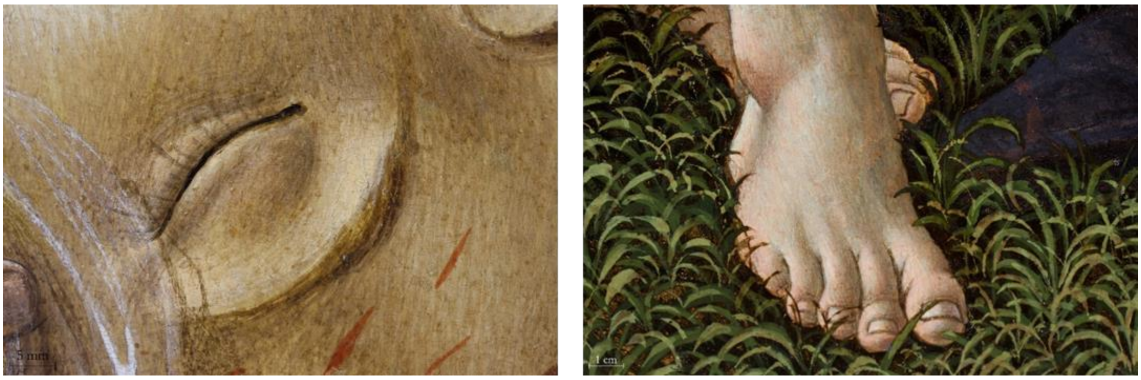

Supplementary Figure 2. The flesh, details of Christ’s head (left) and St. John’s feet (right) show the typical egg tempera layer build-up and paint application by hatching. In contrast, the grass foreground (including the dark green (almost black) paint layer) shows typical properties of oil paints. Pictures: © Wibke Neugebauer, Munich.

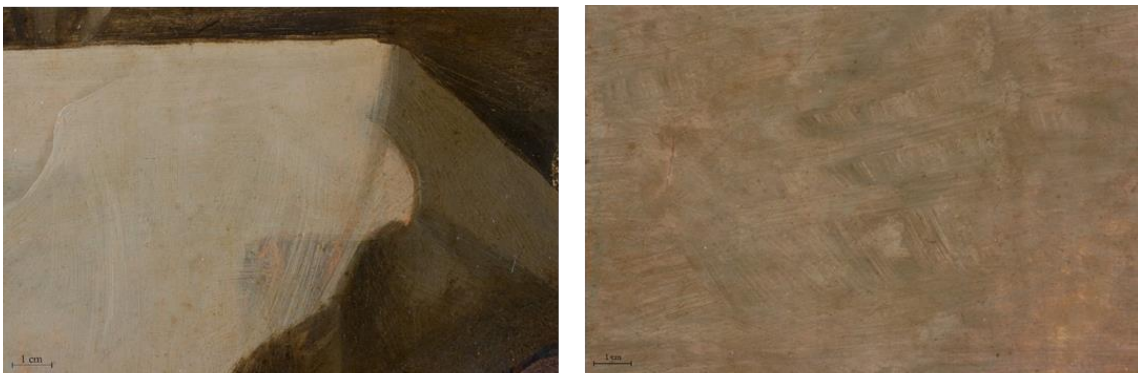

Supplementary Figure 3. Two details of the stone background of the tomb in Botticelli's *Lamentation of Christ* show wet-in-wet mixing of paints, typical for oil painting, which allows a fast and efficient painting process, creating varying, vivid surface impressions of stone in one painting step. Pictures: © Wibke Neugebauer, Munich.

2. Wet paint rheology

Material preparation

Paints preparation*:*

- *Oil paint* preparation consisted of grinding the pigment with linseed oil on a glass plate with a glass muller for 10 min.

- For a *capillary suspension (CapS)*, an oil paint was prepared in which a few drops (from 1 to 4 vol%) of a secondary fluid, either distilled water or fresh egg yolk were added, the paint was then mixed with a palette knife for 1 min, resulting in a very stiff paint.

- The *PCP paint* was prepared in several steps: fresh homogenized egg yolk was diluted with distilled water, which was then ground with the pigment for 10 minutes, and left to dry (c. 30 min at 30 °C). When the water was evaporated, all non-volatile EY components must be deposited on the surface of the pigment particles. The existence of a protein layer on the surface of the pigments was confirmed by scanning electron microscopy images. This protein surface layer was included when calculating φ for the *PCP* paints. Then, the pigment coated with dried egg yolk was ground again with the linseed oil for 10 min. The egg yolk solids were quantified gravimetrically.

A movie for the paint preparation is included with the submitted publication (Supplementary Movie 1).

Experimental methods

Rheological measurements: all rheological measurements were performed on a stress-controlled rheometer (Physica MCR 301, Anton Paar GmbH, Germany) at 20 °C. A three min waiting period was included before starting the measurement to ensure structure recovery. Creep experiments to determine the yield stress σ_y were performed using a vane-and-cup fixture (ST10-4V-8.8, diameter 10 mm) and for the steady shear viscosity measurements a plate-plate geometry (diameter 25 mm, gap 0.5 mm) was used. For yield stress measurements, the shear stress was increased between 10^{-2} Pa and 10^{4} Pa and the deformation was recorded during a total measuring time of 600 s, a time scale relevant for the application of artist paints. Preliminary experiments did not show a substantial variation of the extracted σ_y-values with measuring time. The vane-and cup fixture avoids disturbance of yield stress determination by slip phenomena. At stresses below σ_y a linear relationship between deformation γ and

applied shear stress σ is found (Hooke´s law) since the paint behaves like an elastic solid. At a critical stress level the deformation drastically increases and the yield stress σ_y is determined from the γ (σ) -curves according to the tangent intersection method[2] as shown in Supplementary Fig. 4. Particularly, for *CapS* it is known that structural recovery after shear happens quickly (within seconds) and thixotropic phenomena are of minor relevance[3].

For steady shear viscosity measurements, the shear stress was increased between 10^{-2} Pa and 10^4 Pa and the viscosity was recorded during a total measuring time of 1,340 s. The results shown were determined from at least three measurements of freshly prepared paints, the displayed data are average values and the standard deviations are shown as error bars.

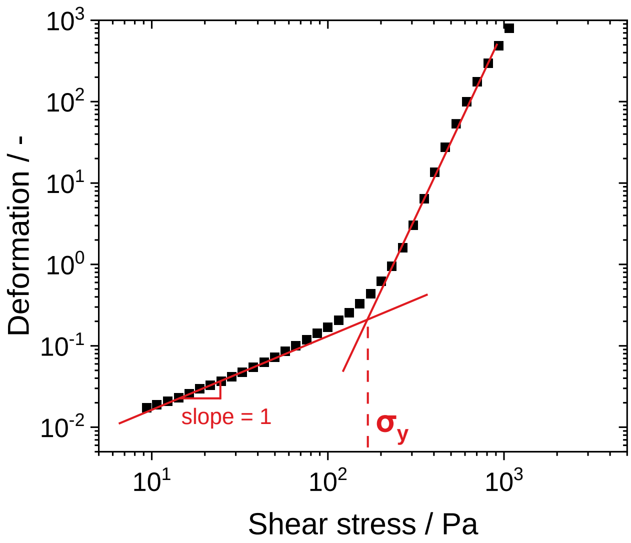

Supplementary Figure 4. Yield stress determination with the tangent intersection point method of a LW PCP paint sample (φ = 42 vol% with 6 vol% of EY).

Brush strokes analysis: Automated paint brush strokes on painting paper (Fabriano, Hot Press, 25% Cotton 300 g/m³) were performed with a universal testing machine (Texture Analyzer TA.XT Plus, Stable Micro Systems, UK) with a custom-made paint brush holder keeping an angle of 60° between the brush (Gussow Oil Painting Brush, 15 mm width, flat, Gerstaecker, Germany) and the painting surface. Brush strokes of 20 x 150 mm were performed in triplicates at a speed of 20 mm/s.

Confocal laser microscopy: automated paint brush strokes profiles of fresh paints were analyzed with a 3D confocal laser microscope with a magnification of 10x (Keyence VK-X100, Japan) to determine the roughness Rz of the paint brush strokes. The single image size of the LSM had a size of 1406 µm x 1054 µm (x- and y-dimension, with a resolution of 0.05 µm), and 24 to 25 images were acquired per cross-section. The resolution in z-direction perpendicular to the image was 0.02 µm.

Contact angle measurements were performed according to the norm ISO 19403-2:2017 using the sessile drop method (DataPhysics Instruments GmbH, OCA15, Filderstadt, Germany). Previously dried pigments were placed as a thin layer on a double-side tape on a flat surface on which was deposited a drop of liquid. For the egg yolk measurements, a thin layer of diluted egg yolk was placed on a glass slide and let to dry prior to deposit a drop of liquid. The value of the contact angle for each liquid is calculated as the arithmetic mean of the measured values.

Particle size distribution as determined through Fraunhofer diffraction (Helos H0309; Sympatec GmbH, Clausthal-Zellerfeld, Germany) using water for particle dispersion in an ultrasonic wet dispersing unit (Quixel and Cuvette, Sympatec GmbH) for both UB and LW pigments.

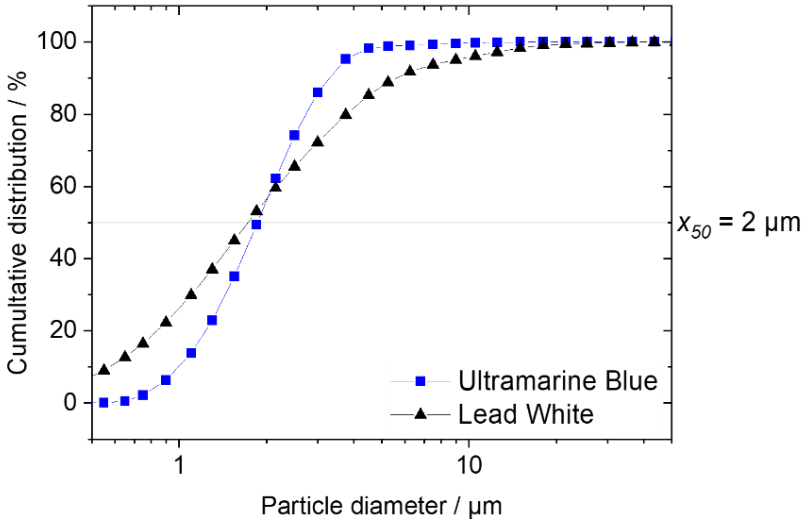

Supplementary Figure 5. Particle size distribution of LW and UB pigments.

Artificial ultramarine blue pigment was used instead of natural ultramarine. Of course for Old Masters, natural ultramarine was the only available source of this pigment. Highly heterogeneous particles (some of which are very large, much more than 10 µm in diameter) typically present in natural ultramarine, however, would have disturbed rheological measurements, and would have added a higher degree of variability in the experimental parameters.

Scanning electron microscope (SEM) pictures were performed using LEO1530 microscope (Carl Zeiss AG, Oberkochen, Germany) for both UB and LW pigments.

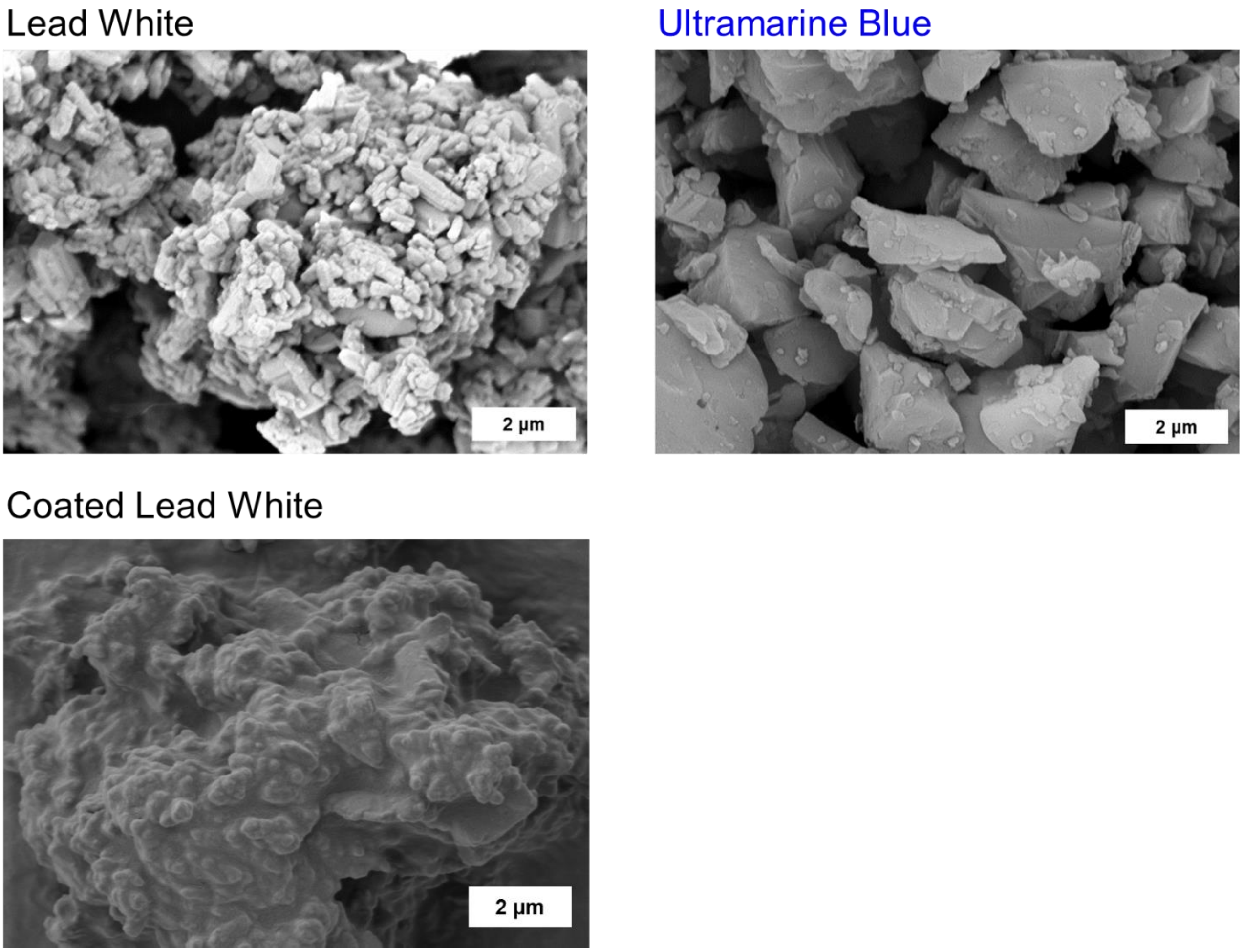

Supplementary Figure 6. Scanning electron microscope (SEM) pictures of LW and UB pigments (above) and of LW coated with a ≈ 1 µm egg yolk layer (below).

X-ray diffraction (XRD): An X'Pert Pro PANalytical diffractometer equipped with an X'Celerator detector with a Cu X-ray tube (λ = 1.54 Å) was used with a Ni-filtered Cu–Kα radiation source. The X-ray tube was operated at 40 kV and 30 mA. The diffraction patterns were collected under the following conditions: 2θ range 3–70°, step size 0.02°. Soller and anti-scatter slits were used on the incident and diffracted beams; a divergent slit was used on the incident beam. A zero-background sample stage was used. The phase identification of the samples was performed using the X'Pert HighScore programand the ICCD database. Few mg of UB and LW pigments were analysed with Xray Diffraction technique.

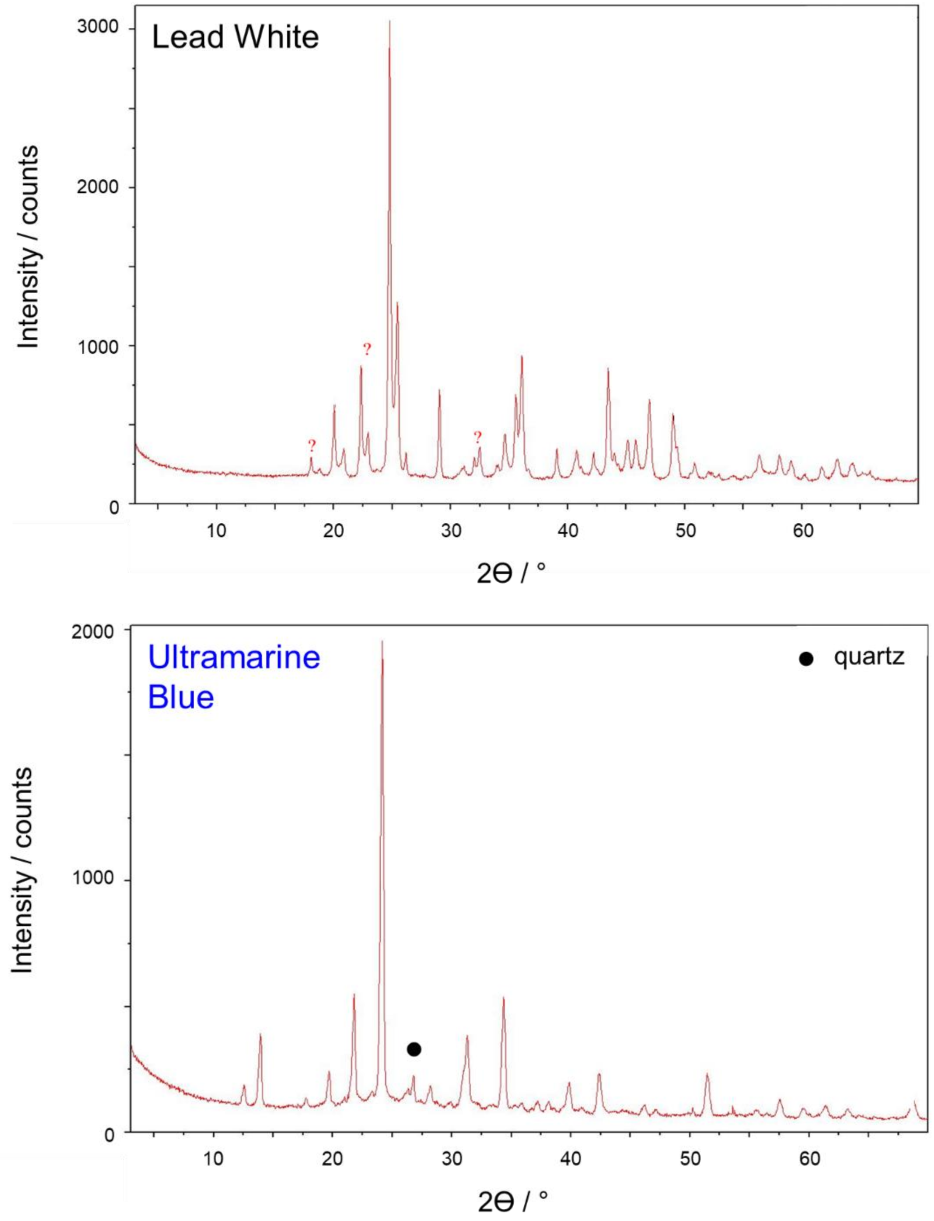

Supplementary Figure 7. XRD patterns of LW (above) and UB (below) pigments used for this study.

In LW, the main crystalline phase is cerussite, a lead carbonate. Traces of other unidentified crystalline phases, not related to other lead compounds (i.e hydrocerussite, plumbonacrite, lead acetate, lead oxides) are also present (corresponding peaks are marked by **?**).

In UB, all peaks refer to the crystalline phase of synthetic ultramarine blue, a sodium aluminum silicate containing sulfur, with the exception of the peak at 2Θ 26,70°, which is related to quartz (SiO_2, marked with the circle).

Supplementary Table 2. Contact angle measurements according to ISO 19403-2:2017

Contact angle / °	Dried EY	Lead White	Ultramarine Blue
Linseed Oil	38 ± 7	9 ± 1	9 ± 2
Water	29 ± 7	84 ± 3	55 ± 6

Supplementary Table 3. Interfacial tension measurements according to ISO 19403-3:2017

Interfacial tension / mN/m	Water	Liquid EY
Linseed Oil	10.8 ± 0.7	1.9 ± 0.3

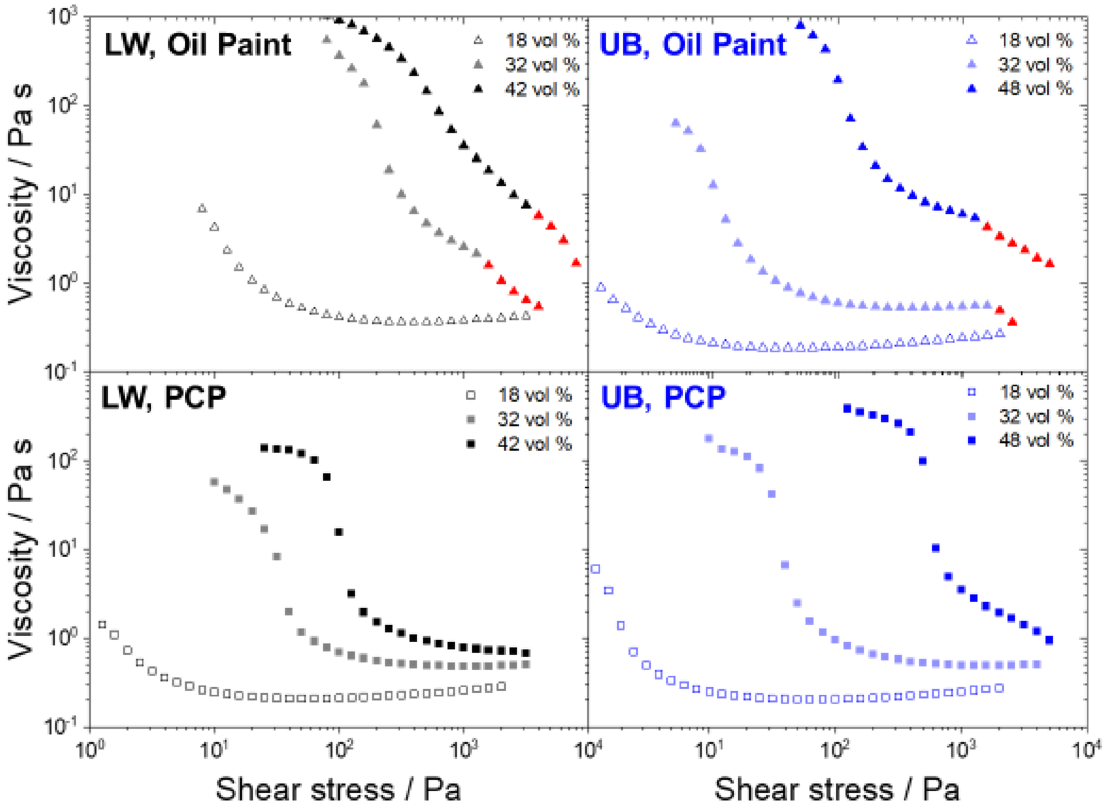

Supplementary Figure 8. Viscosity measurements as a function of the shear stress of lead white LW (black) and ultramarine blue UB (blue) oil paints (▲) and pigment coated paints PCP (■) at different solids content. In red are displayed the values obtained due to the spillage of the paint in plate-plate geometry.

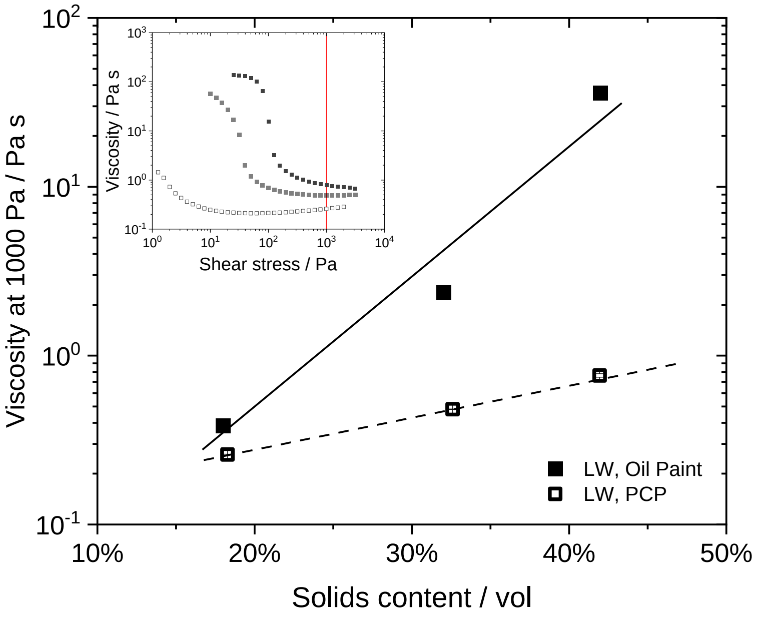

Supplementary Figure 9. Viscosity measurements at 1000 Pa of LW oil paints (■) and LW PCP (□) as a function of the solids content. Coating the pigment with an egg yolk layer reduces the viscosity of LW paints at high shear, hence allows for better brushability at high pigment loading. Similar results were obtained for ultramarine blue pigment paints (data not shown).

We measured the viscosity at high shear stresses well above the yield stress of paints with increasing solids content, prepared with or without pigment coating. The viscosity measurements of paints prepared with the raw pigments dispersed in the oil showed spillage at high shear stresses (red symbols in Supplementary Fig. 8). When the pigments are coated with egg yolk, the high shear regime is accessible and the data show that the increase in high shear viscosity is much less pronounced for the PCP systems than for the oil paints (Supplementary Fig. 9).

3. From wet paints to solid films

Experimental methods

Gravimetric measurements: fresh paint samples were poured in circular custom-made aluminum sample holders (diameter 25 mm, thickness 0.4 mm) and weighted regularly upon time. The weight of each sample was normalized to the oil content of the fresh paint for comparison.

Texture analysis: the change of hardness of the paint sample films upon time were performed with a universal testing machine (Texture Analyzer TA.XT Plus, Stable Micro Systems, UK) equipped with a stainless steel tip (diameter 8 mm) and a custom-made sample holder plate (405 x 15 mm), allowing to keep the thickness of the paint layer at 0.4 mm. The tip penetrated into the sample at 10 mm/s at a depth of 0.3 mm from the paint surface. Each experiment was performed in triplicate, the displayed data are average values and the standard deviations are shown as error bars. Alongside with the determination of the hardness (which is the force necessary to penetrate the paint layer divided by the surface of the tip), the sample was considered as "dry-to-touch" by visual control of its surface (see Supplementary Fig. 7B).

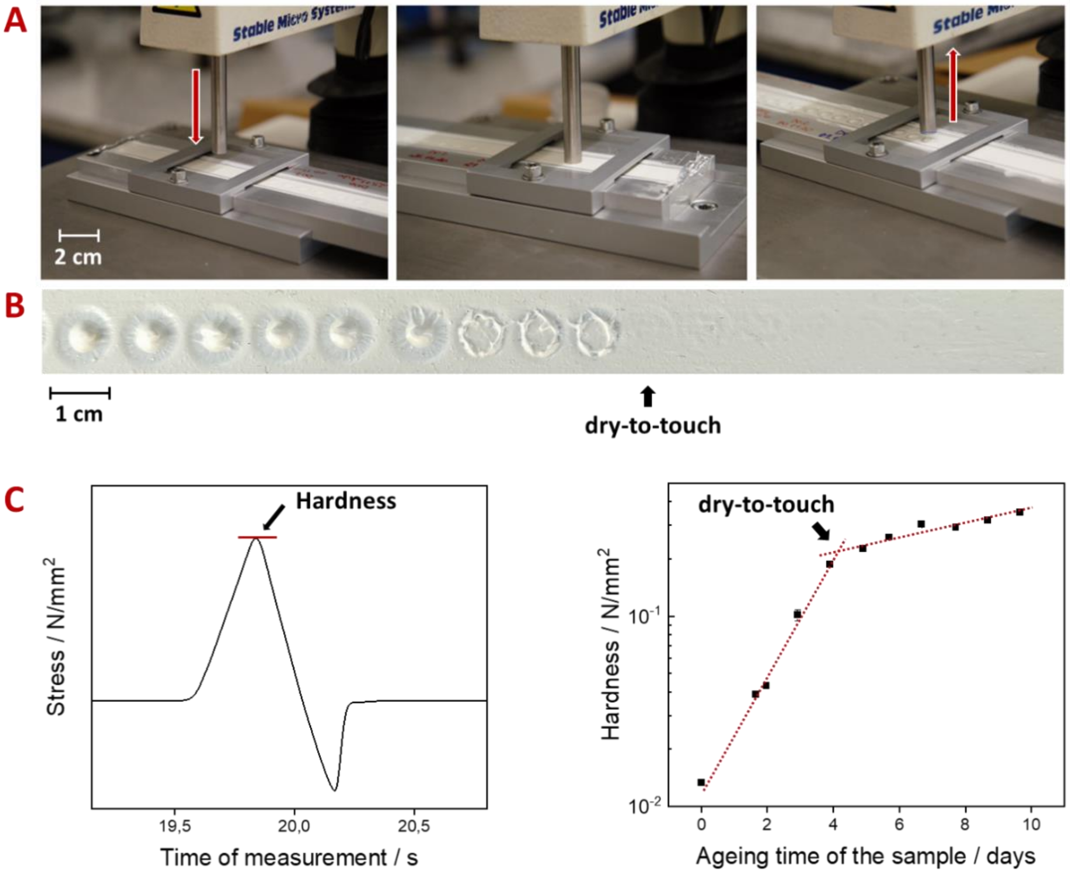

Supplementary Figure 10. (A) Custom-made equipment on a universal testing machine to determine the "dry-to-touch" state of the paints. (B) Traces left at the paint surface after measurements in triplicates, useful for the visual control of the "dry-to-touch" state. (C) Experimental determination of the hardness of a paint (left) and "dry-to-touch" determination from the hardness of the paint layer upon natural ageing (right).

Humidity storage: pigments were ground prior to be disposed on thin layers (around 2 mm) in aluminum containers in desiccators containing saturated solution of salts ($MgCl_2$ or NaCl) or drying medium (silica gel) during 14 days. Relative humidity at T = 20.0 ± 0.5 °C was determined with a temperature and humidity sensor (Digi-Sense™ USB Datalogger, Cole-Parmer). The moisture taken up by the pigments was determined gravimetrically as follows: the weight of the samples after 2 weeks storage in desiccators containing saturated solution of salts ($MgCl_2$ or NaCl) or drying medium (silica gel) was subtracted from the weight of the pigments stored at laboratory conditions (T = 20.0 ± 0.5 °C) before storage in desiccators.

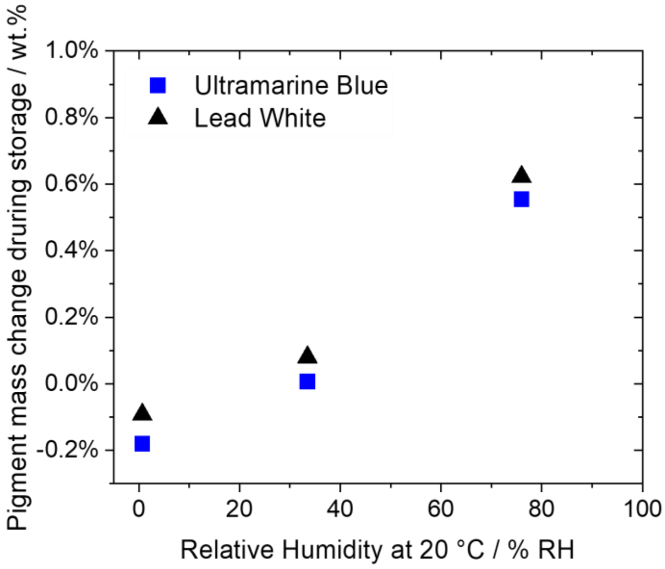

Supplementary Figure 11. Pigment mass change after 2 weeks of storage at different humidity conditions at 20 °C.

Differential Scanning Calorimetry *(DSC)* analyses were performed with a differential scanning calorimeter (TA Instruments DSC 250) under constant nitrogen flow (80 mL/min) in the 25−250 °C temperature range, with a 10 °C/min heating rate. Paint samples (1−3 mg) were scratched from the cast of paint disposed on glass slides and placed in aluminum pans, then sealed. Since the oil is the only component susceptible to exhibit thermal effect in oil paints in the studied temperature range, DSC curves were normalized to the oil content. Temperature and heat flow were calibrated with indium, and the baseline was calibrated using two empty aluminum pans.

Thermogravimetric analyses (TG) were carried out with TA-Instruments thermo-balance, model Q5000IR, at constant nitrogen flow (25 mL/min) in the 25−900 °C temperature range (25-300 °C for LW-containing samples) at a heating rate of 20 °C/min. Paint samples (ca. 3 mg) were scratched from the cast of paint disposed on glass slides and placed in platinum pans (and in aluminum crucibles in on the platinum pans for LW-containing samples).

Isothermal thermogravimetric analyses were performed on the same instrument for recording the oxygen uptake curve under a constant air flow (25 mL/min). The freshly prepared samples (ca. 7-19 mg) were placed in platinum crucibles and analyzed at 80 °C for 5,500 min. Temperature calibration was based on the Curie point of paramagnetic metals (Alumel, Ni, $Ni_{83\%}Co_{17\%}$, $Ni_{63\%}Co_{37\%}$, $Ni_{37\%}Co_{63\%}$).

Pyrolysis coupled with mass spectrometry (Py/GC/MS): the instrumentation consisted of a microfurnace multishot pyrolyzer EGA/Py-3030D (Frontier Lab) coupled to a gas chromatograph 6890 N (Agilent Technologies, Palo Alto, CA, USA) and to an Agilent 5973 Mass Selective Detector. The split/split-less injector was used in split mode at 280 °C, with a split ratio 30:1. The chromatographic conditions were as follows: 50 °C isothermal for 2 min, 10 °C/min up to 280 °C and isothermal for 2 min, and 15 °C/min up to 300 °C and isothermal for 30 min. The carrier gas (He, purity 99.9995%) was used in the constant flow mode at 1.0 mL/min. The temperatures of the MS transfer line, MS ion source, and MS quadrupole were 280, 230, and 150 °C, respectively. The mass spectrometer was operated in EI positive mode (70 eV) with a scan range m/z 50−600. MS spectra were recorded in TIC mode. Ca. 150 µg of the sample were placed in a pyrolysis cup where 4 µL of 1,1,1,3,3,3-hexamethyldisilazane (HMDS) were added as a silylating agent for the in-situ derivatization of pyrolysis products. The temperature of the furnace for pyrolysis was set at 550 °C.

Fourier-Transformed Infrared Spectroscopy (TRANS-FTIR): Infrared spectra were recorded by using a Perkin-Elmer Spectrum 100 FTIR Spectrophotometer (Perkin Elmer, USA), equipped with a universal attenuated total reflectance (ATR) accessory and a triglycine sulphate TGS detector. To monitor the changes in the protein structure as well as the lipid oxidation upon time, fresh paint thin layers were applied on BF_2 windows (diameter 13 mm) and regularly analysed by direct transmittance spectroscopy (TRANS-FTIR) after defined periods of time. For each

sample, 128 scans were recorded in the range of 4,000-600 cm^{-1}, averaged, and Fourier-transformed to produce a spectrum with a nominal resolution of 4 cm^{-1}. The amide I peak (1,600-1,700 cm^{-1}) was fitted with a frequency deconvolution procedure according to the method described in *Bramanti et al. (1994)*[4,5].

I. Gravimetric and thermogravimetric analyses (TG) measurements upon time

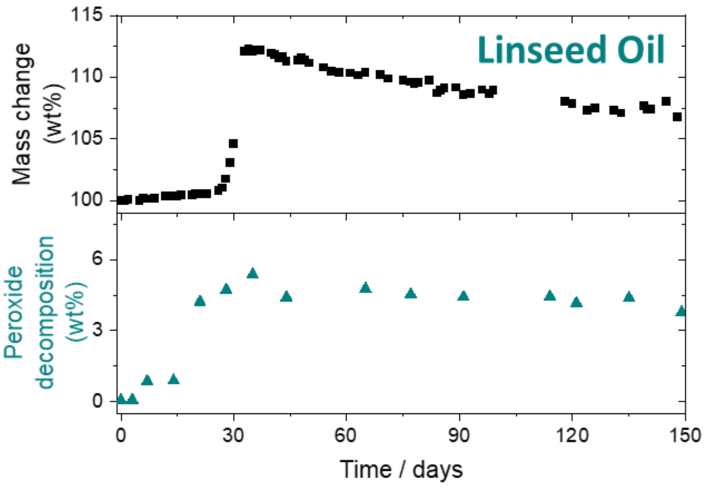

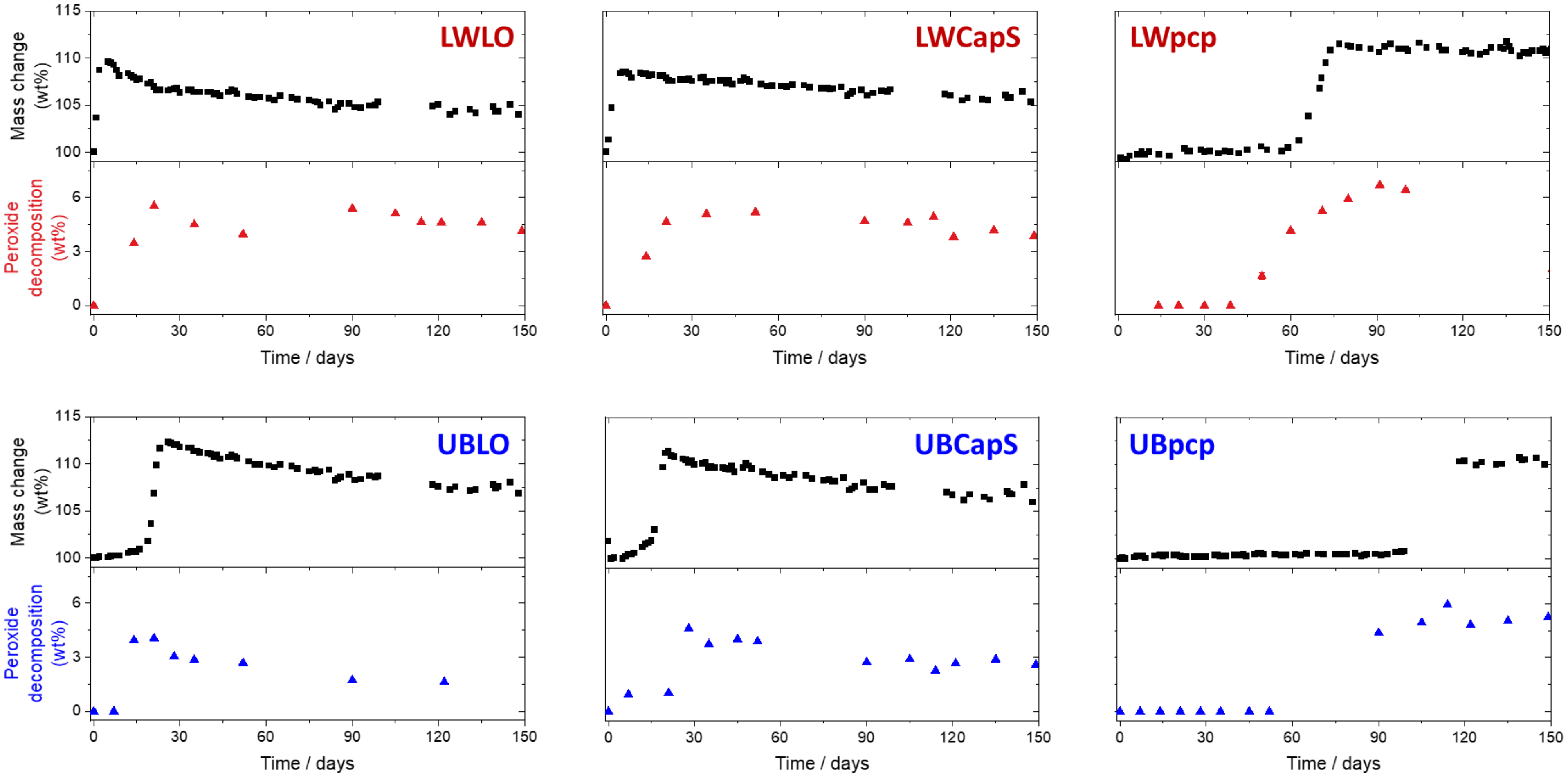

Supplementary Figure 12. Comparison of the mass increase of linseed oil-based samples (above) and the associated peroxide decomposition performed by TG measurements (below, at T = 135-180 °C depending on the paint composition), all normalized to the oil content. Solids content of LW LO and LW PCP are φ = 31 and 29 vol% respectively, including 15 vol% (PCP) of EY. Solids content of UB LO and UB PCP are φ = 6 and 16 vol% respectively, including 12 vol% (PCP) of EY. Solids content of LW CapS and UB CapS are φ = 31 and 33 vol% respectively, including 2 (LW) and 3 vol% (UB) of EY. PCP stands for protein coated pigment paint, CapS for capillary suspension paint and LO for oil paint.

II. Differential scanning calorimetry (DSC) measurements upon time

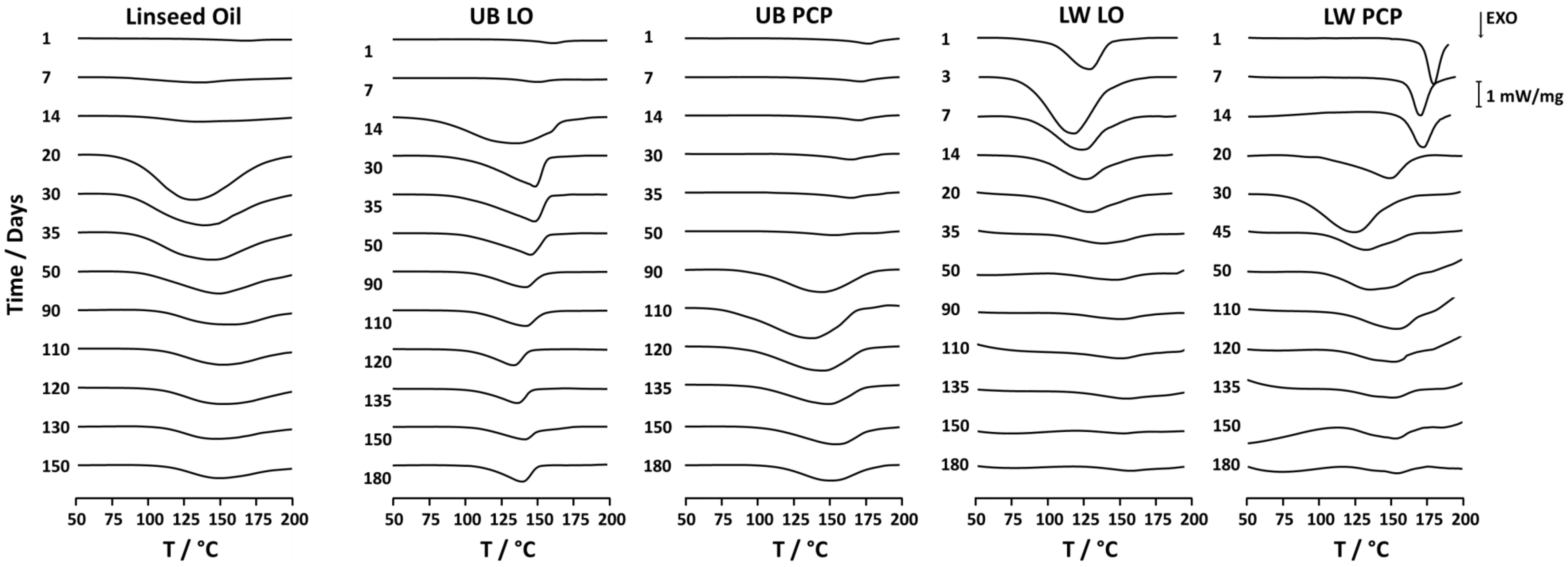

Supplementary Figure 13. DSC curves (normalized to the oil content) of raw linseed oil and mock-up paints exposed to natural ageing. Solids content of LW LO and LW PCP are φ = 31 and 29 vol% respectively, including 15 vol% (PCP) of EY. Solids content of UB LO and UB PCP are φ = 6 and 16 vol% respectively, including 12 vol% (PCP) of EY. PCP stands for protein coated pigment paint and LO for oil paint.

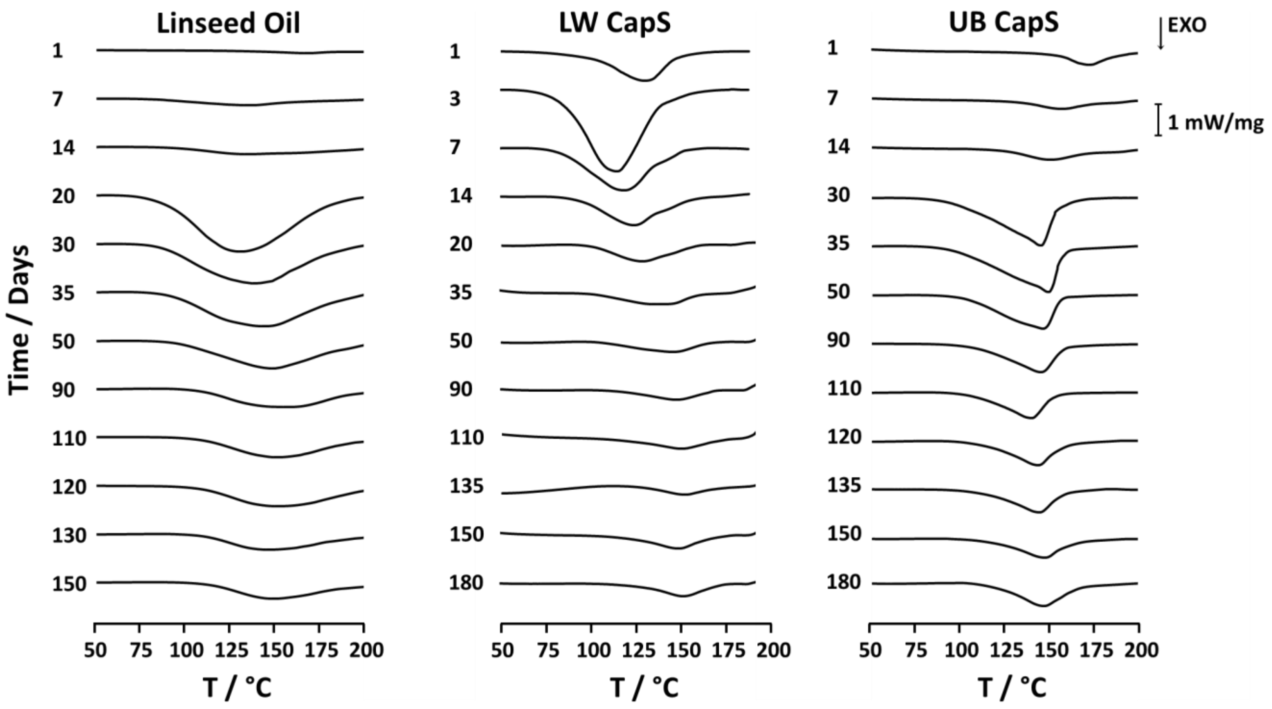

Supplementary Figure 14. DSC curves (normalized to the oil content) of linseed oil and mock-up capillary suspension paints CapS exposed to natural ageing. Solids content of LW CapS and UB CapS are φ = 31 and 33 vol% respectively, including 2 (LW) and 3 vol% (UB) of EY.

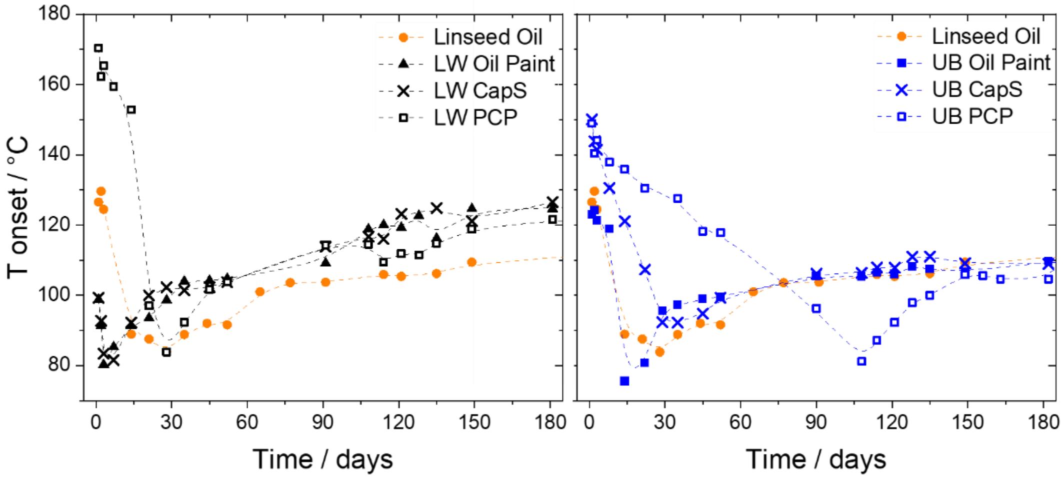

Supplementary Figure 15. Onset temperature of the overall exothermic peak associated to the decomposition of peroxides and hydroperoxides (endothermic) and the recombination of radical species (exothermic) as a function of the evaluation time. Solids content of LW LO, LW CapS and LW PCP are φ = 31, 31 and 29 vol% respectively, including 2 (CapS) 15 vol% (PCP) of EY. Solids content of UB LO, UB CapS and UB PCP are φ = 6, 33 and 16 vol% respectively, including 3 (CapS) and 12 vol% (PCP) of EY. PCP stands for protein coated pigment paint, CapS for capillary suspension paint and LO for oil paint.

Supplementary Table 4. Onset temperature of the normalized DSC curves for linseed oil (LO). Solids content of LW LO and LW PCP are φ = 31 and 29 vol% respectively, including 15 vol% (PCP) of EY. Solids content of UB LO and UB PCP are φ = 6 and 16 vol% respectively, including 12 vol% (PCP) of EY.

**Time / days**	**T onset / °C**				
	Linseed Oil	UB LO	UB PCP	LW LO	LW PCP
**1**	127	123	149	99	170
**2**	130	124	140	91	162
**3**	124	121	144	80	165
**7**	78	119	138	85	159
**14**	89	76	136	91	153
**20**	88	81	130	94	97
**30**	84	96	134	99	84
**35**	89	97	128	104	92
**50**	92	100	118	105	104
**90**	104	105	96	109	114
**110**	106	105	81	119	114
**120**	105	106	92	119	112
**135**	106	108	100	116	115
**150**	109	107	106	125	119
**180**	110	110	105	125	122

Supplementary Table 5. Onset temperature of the normalized DSC curves for linseed oil (LO). Solids content of LW CapS and UB CapS are φ = 31 and 33 vol% respectively, including 2 (LW) and 3 vol% (UB) of EY.

Time / days	T onset / °C		
	Linseed Oil	LW CapS	UB CapS
**1**	127	99	150
**2**	130	93	144
**3**	124	83	141
**7**	78	82	131
**14**	89	92	121
**20**	88	100	107
**30**	84	102	92
**35**	89	101	92
**50**	92	104	99
**90**	104	114	106
**110**	106	117	106
**120**	105	123	108
**135**	106	125	111
**150**	109	121	109
**180**	110	127	109

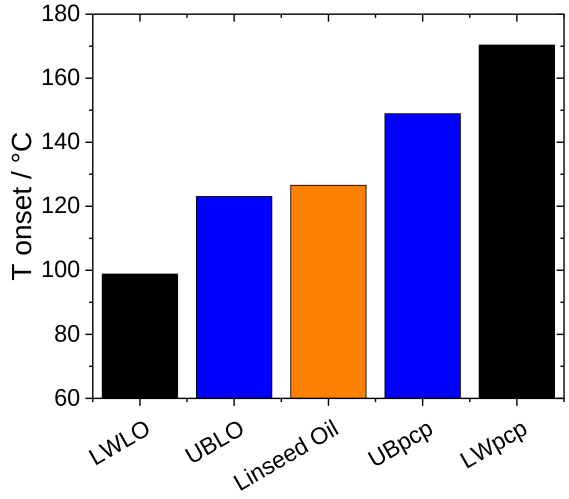

Supplementary Figure 16. Onset temperature of the overall exothermic peak on the first day of natural ageing. Solids content of LW LO and LW PCP are φ = 31 and 29 vol% respectively, including 15 vol% (PCP) of EY. Solids content of UB LO and UB PCP are φ = 6 and 16 vol% respectively, including 12 vol% (PCP) of EY. PCP stands for protein coated pigment paint and LO for oil paint.

III. Py/GC/MS analyses display the building of a polymeric network, delayed by coating the pigment with a proteinaceous layer

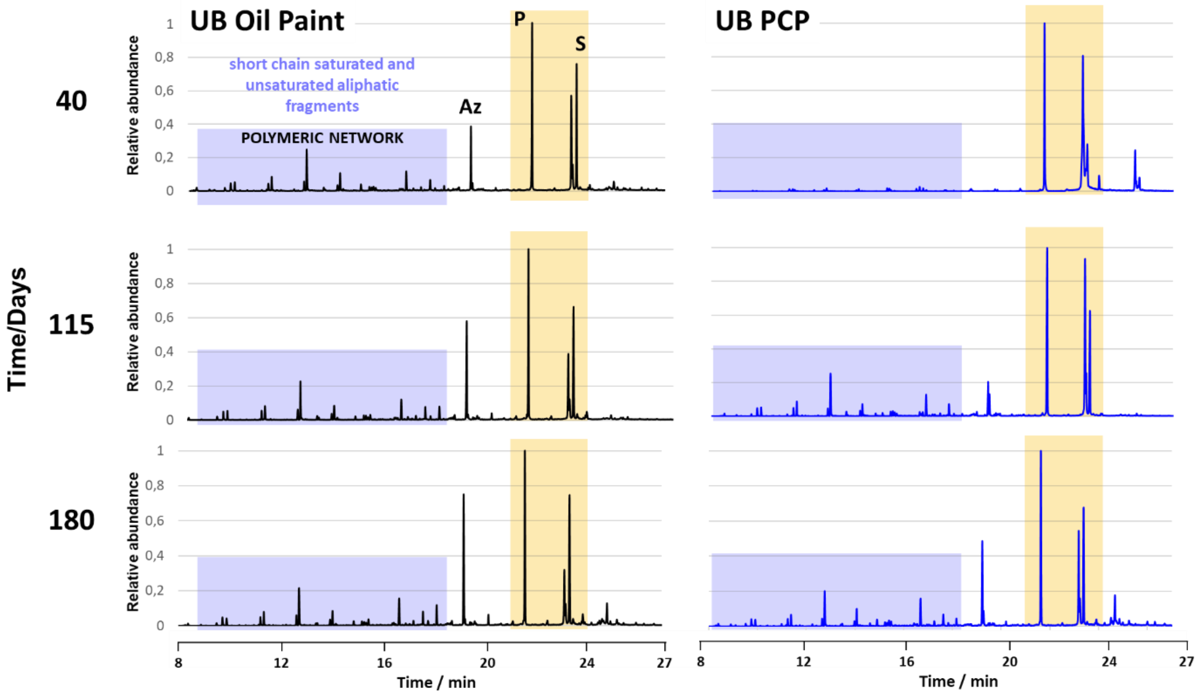

Supplementary Figure 17. Extracted ion pyrograms of m/z 129 of model oil paintings after 40, 115 and 180 days of natural aging. Solids content of UB oil paint and UB PCP are φ = 6 and 16 vol% respectively, including 12 vol% (PCP) of EY. All the paints have been normalized to the palmitic acid peak (P). (S) represents the stearic acid and (Az) the azelaic acid peak. The evolution of the polymeric network upon time is visible in the blue rectangle, more details can be found in Fig. 5 in the main manuscript (see text for an explanation).

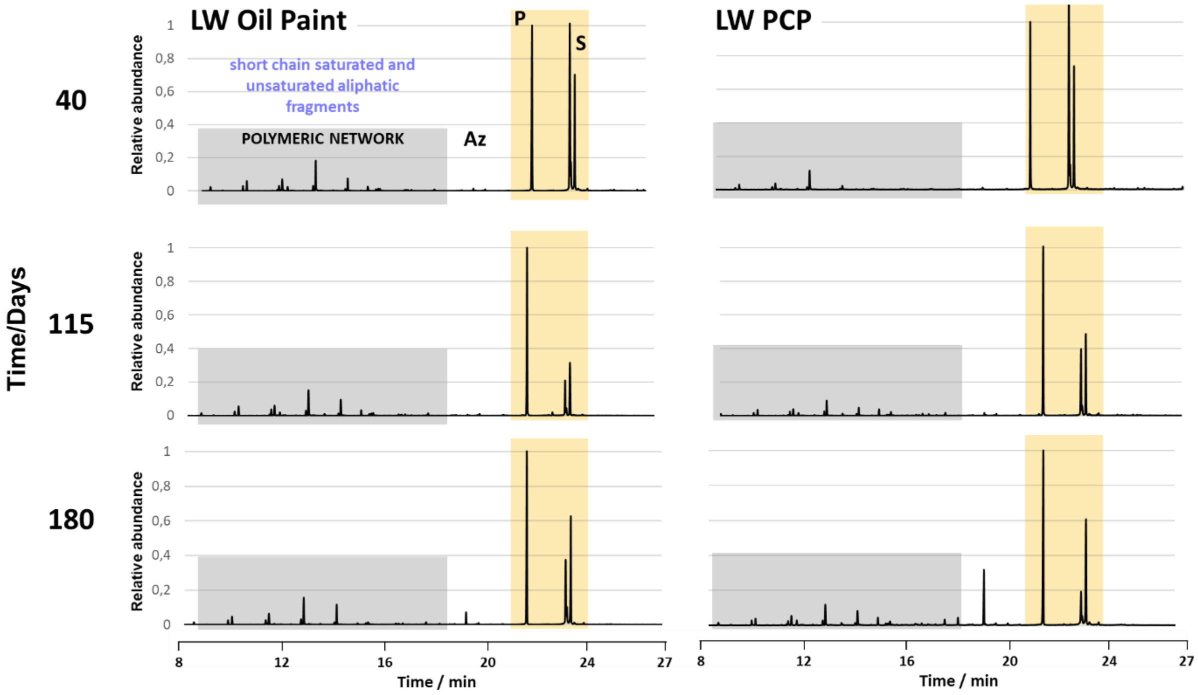

Supplementary Figure 18. Extracted ion pyrograms of m/z 129 of model oil paintings after 40, 115 and 180 days of natural aging. Solids content of LW oil paint and LW PCP are φ = 31 and 29 vol% respectively, including 15 vol% (PCP) of EY. All the paints have been normalized to the palmitic acid peak (P). (S) represents the stearic acid and (Az) the azelaic acid peak. The evolution of the polymeric network upon time is visible in the grey rectangle, more details can be found in Fig. 5 in the main manuscript (see text for an explanation).

IV. Oxygen uptake at 80 °C by TG

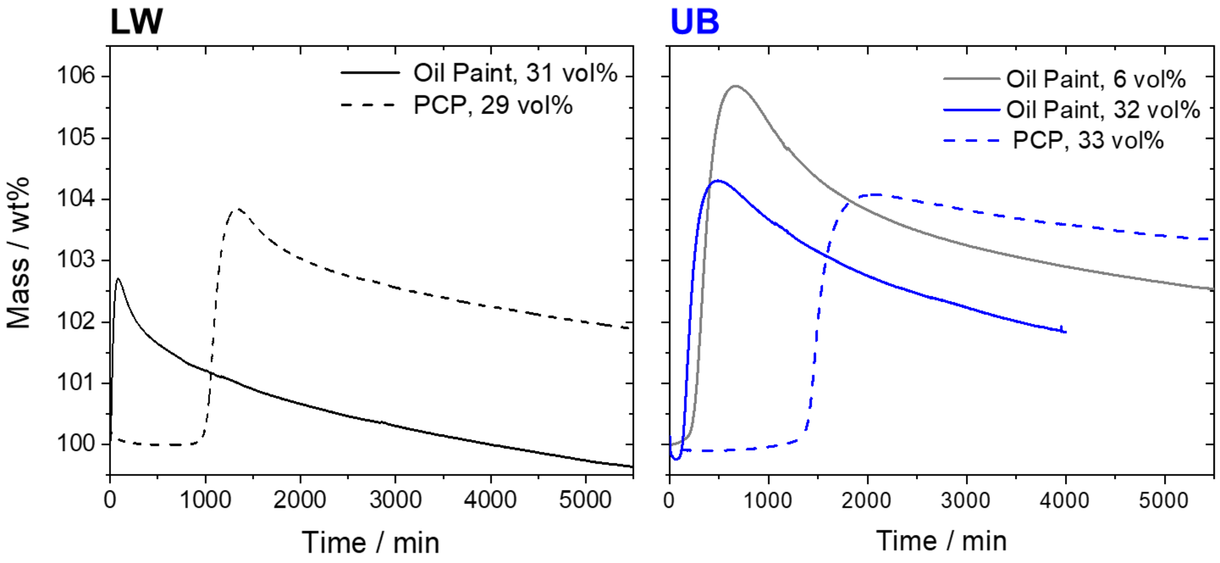

Supplementary Figure 19. Oxygen uptake experiments in TG in accelerated conditions at 80 °C. Solids content of UB oil paint and LW oil paint is only composed on pigments. Solids content of UB protein coated paint (PCP) is composed of 21 vol% of UB and 12 vol% of egg yolk and LW PCP is composed of 14 vol% of LW and 15 vol% of egg yolk.

V. TRANS-FTIR protein conformation analyses and mass changes of pigment coated paints samples upon time

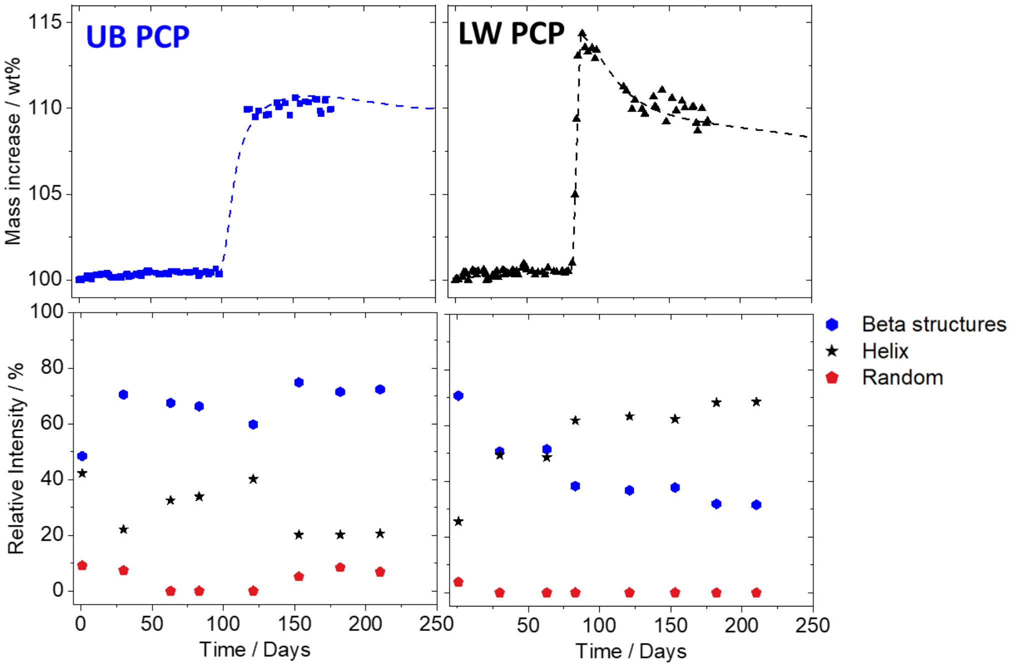

Supplementary Figure 20. Above: mass increase normalized to the oil content of UB protein coated paint PCP (blue) and LW PCP (black) as a function of time. Below: relative intensity % of the optical density of the peaks assigned to beta structures, helix and random coils, obtained by fitting the amide I band of TRANS-FTIR spectra of the PCP samples as a function of time. Solids content of UB PCP is φ = 33 vol%, including 12 vol% of EY and LW PCP is φ = 29 vol%, including 15 vol% of EY.

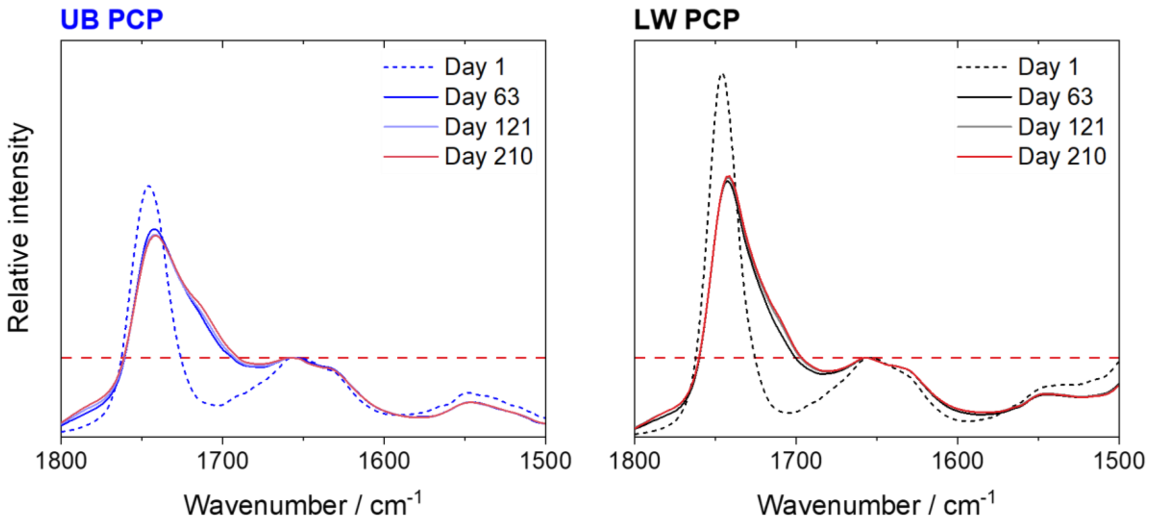

Supplementary Figure 21. TRANS-FTIR spectra of UB PCP (blue) and LW PCP (black) paints at 1, 63, 121 ans 210 days of natural ageing. Solids content of UB PCP is φ = 33 vol%, including 12 vol% of EY and LW PCP is φ = 29 vol%, including 15 vol% of EY.

Supplementary Table 6. Peak attribution according to Bramanti et al.(1996)[5]

Helixes		β-structures		Random	
1658 cm^{-1}	α-helix	1610 cm^{-1}	intermolecular β-sheets	1644-47 cm^{-1}	Random coil
1667 cm^{-1}	extended helix	1615 cm^{-1}	anti-parallel β-sheets		
		1630 cm^{-1}	β-turns		
		1677 cm^{-1}	anti-parallel β-turns		
		1681-91 cm^{-1}	anti-parallel β-sheets		

We followed the mass change of egg-based samples upon time on aluminum pans and the modification of the protein conformation on BF_2 pellets to understand how the conformation changes of egg proteins are related to the curing of the linseed oil.

When egg yolk coats the UB pigment prior to be dispersed into the oil, the proteins are present mostly β-structures (≈ 70 %), helices (≈ 30 %) at less extent and no random conformation. The oil undergoes curing reactions at around 100 days of ageing, causing no major changes of the secondary structure of the polypeptide chain upon curing.

In LW PCP, the proteins change their conformation in correspondence with the mass uptake of the oil (from 50% helix and 50% β-structures with the “fresh” oil to a majority of helicoidal structures (≈ 65 %) over β-structures when the oil undergoes curing reactions). This change of protein structure upon curing suggests that the egg proteins interact with the LW pigment upon the oil curing, provoking a modification in the secondary structure of the polypeptide chain, leading to a reorganization of the polymeric network in the paint.

4. Wrinkling

VI. Example of wrinkling in artists' painting

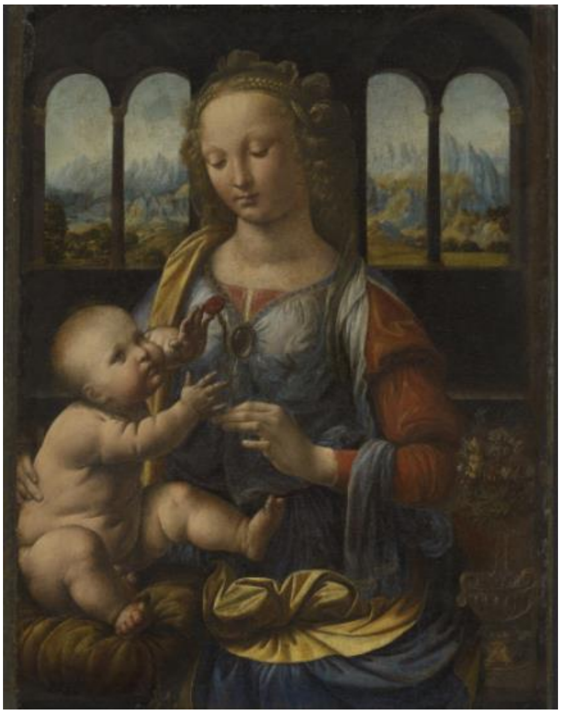

Supplementary Figure 22. Leonardo da Vinci, *Madonna of the Carnation*, c. 1475, 62 x 48,5 cm, Inv. No. 7779, © Bavarian State Painting Collections, Munich.

This artwork painted by Leonardo da Vinci shows wrinkling in the shadows of the flesh paint of Mary and the child shown in Fig. 7a of the main text. Unfortunately the paints in the flesh could not be sampled and therefore no discussion of the paint composition is possible.

VII. Increasing the solids fraction in oil paints prevents the formation of wrinkles

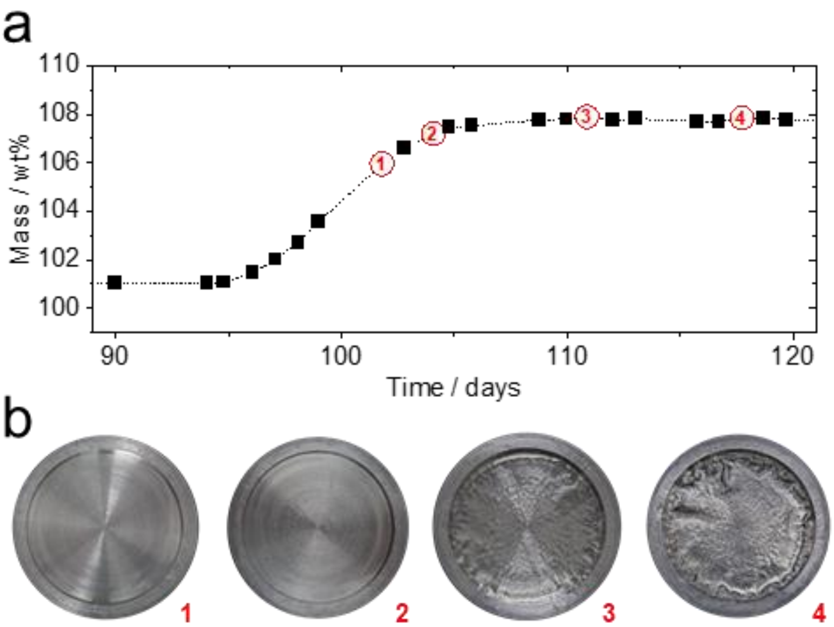

Supplementary Figure 23. **a** Mass increase of raw linseed oil at a thickness layer of 1.0 mm and **b** corresponding photographs of the wrinkling at the time indicated.

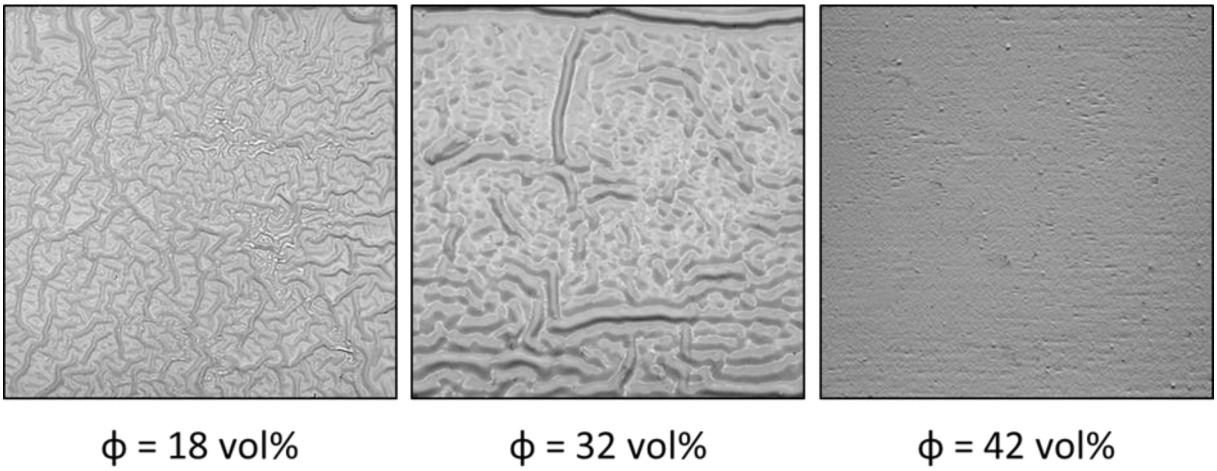

Supplementary Figure 24. Surface of LW oil paint samples (thickness 1.0 mm) at increasing pigment content after several months of natural ageing.

Supplementary References

1. Dietemann, P., Fischer, U., Karl, D., Baumer, U. & Steuer, C. Die Bindemittel der Florentiner Malerei. in *Florentiner Malerei – Alte Pinakothek. Die Gemälde des 14. bis 16. Jahrhunderts* (eds. Schumacher, A., Kranz, A. & Hojer, A.) 92–105 (2017).
2. Yüce, C. & Willenbacher, N. Challenges in rheological characterization of highly concentrated suspensions — A case study for screen-printing silver pastes. *J. Vis. Exp.* **2017**, 1–17 (2017).
3. Koos, E., Kannowade, W. & Willenbacher, N. Restructuring and aging in a capillary suspension. *Rheol. Acta* **53**, 947–957 (2014).
4. Bramanti, E., Bramanti, M., Stiavetti, P. & Benedetti, E. A frequency deconvolution procedure using a conjugate gradient minimization method with suitable constraints. *J. Chemom.* **8**, 409–421 (1994).
5. Bramanti, E. & Benedetti, E. Determination of the secondary structure of isomeric forms of human serum albumin by a particular frequency deconvolution procedure applied to Fourier transform IR analysis. *Biopolymers* **38**, 639–653 (1996).
