# Supplementary material for: A holistic view on the role of egg yolk in Old Masters’ oil paints
Source: Nat Commun. 2023 Mar 28;14:1534. doi: 10.1038/s41467-023-36859-5 (PMC10050151; doi:10.1038/s41467-023-36859-5)
Supplement: Supplementary file 2 — Description of Additional Supplementary Files [file 41467_2023_36859_MOESM2_ESM.pdf]

Description of Additional Supplementary Files

File Name: Supplementary Movie 1
Description: Preparation of an oil paint and a capillary suspension (CapS) paint.
